# Dual Role of Exosomes in Parkinson's Disease: Adenine Exerts a Beneficial Effect

**DOI:** 10.1111/cns.70331

**Published:** 2025-04-16

**Authors:** Lei Chen, Yi‐Ting Shao, Ji Geng, Hua Liu, Qing‐Shan Liu, Yong Cheng, Ting Sun

**Affiliations:** ^1^ Key Laboratory for Ethnomedicine for Ministry of Education, Center on Translational Neuroscience, School of Pharmacy Minzu University of China Beijing China; ^2^ Institute of National Security Minzu University of China Beijing P. R. China; ^3^ Institute of Medicinal Biotechnology Chinese Academy of Medical Sciences and Peking Union Medical College Beijing P. R. China

**Keywords:** adenine, exosome, metabolite, Parkinson's disease, pathogenicity, therapeutic agent

## Abstract

**Aims:**

Developing validated treatments for Parkinson's disease (PD) remains a priority for clinicians and researchers. The lack of viable therapies may stem from an incomplete understanding of PD pathogenesis and inadequate therapeutic candidates. The production and transmission of exosomes are gaining recognition in the pathogenesis of neurodegenerative diseases. However, how exosomes affect the pathophysiology of PD has not been well elucidated.

**Methods:**

Here, we investigated the effect of exosomes secreted by rats that were treated with saline or 1‐methyl‐4‐phenyl‐1,2,3,6‐tetrahydropyridine hydrochloride (MPTP) in treating healthy or PD model mice, and we evaluated the efficacy of peripheral and intracranial administration of adenine, which is an exosomal metabolite identified through widely targeted metabolomics.

**Results:**

We found that exosomes derived from the blood of healthy rats alleviated motor dysfunction, dopaminergic neuron loss in the substantia nigra pars compacta and striatum, oxidative injury, and neuroinflammation. Conversely, exosomes from the blood of PD model rats reproduced the behavioral phenotype and pathology of PD in healthy mice. Additionally, peripheral and intracranial administration of adenine ameliorated the motor coordination disorder and dopaminergic neuron loss, and maintained the homeostasis of oxidative stress and neuroinflammation by activating cAMP/PKA signaling in PD.

**Conclusion:**

Together, these findings shed light on the mechanism by which exosomes participate in the pathophysiology of PD by transmitting the metabolite adenine and providing potential therapeutic strategies.

## Graphical abstract

Exosomes derived from the blood of healthy rats have therapeutic effects on pathology in PD mice; conversely, exosomes released by PD model rats cause PD‐like pathological features in healthy mice. Adenine, which is enriched in healthy exosomes, plays a key role in PD pathology by activating the cAMP/PKA signaling pathway.

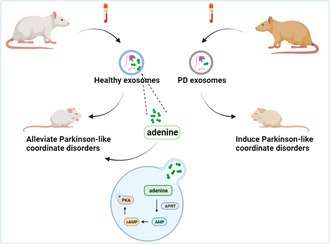

**Abbreviations**
A2AR: adenosine A2A receptor
APRT: adenine phosphoribosyl transferase
cAMP: cyclic adenosine monophosphate
MDA: malondialdehyde
MPTP: 1‐methyl‐4‐phenyl‐1,2,3,6‐tetrahydropyridine hydrochloride
NTA: Nanoparticle tracking analysis
PBS: phosphate‐buffered saline
PD: Parkinson's disease
PKA: protein kinase A
SNpc: substantia nigra pars compacta
T‐AOC: total antioxidant capacity
TEM: transmission electron microscopy
TH: tyrosine hydroxylase

## 1 Introduction

Parkinson's disease (PD), the second most prevalent neurodegenerative disease, poses a significant threat to global health, with millions of patients at risk of losing autonomy and facing reduced life expectancy [1]. With the aging of the global population, the number of patients with PD is predicted to exceed 10 million by 2030 [2]. The clinical features of PD include motor symptoms (static tremors, bradykinesia, rigidity, and postural instability) and nonmotor symptoms (olfactory dysfunction, insomnia, and constipation) [3]. Despite considerable efforts in the field, the current understanding of the pathophysiology of PD remains rudimentary, and there are no validated approaches for managing this condition [4]. Therefore, investigating the etiology of PD is critical for developing effective therapeutics to address the disease in its entirety.

Current research has shown that PD is characterized by the degeneration of dopaminergic neurons in the substantia nigra pars compacta (SNpc), deficiency of dopamine content in the striatum, and the appearance of Lewy bodies [5]. Although the etiology of PD is still far from being understood, many pathophysiological changes are thought to be involved in the development of PD, including neuroinflammation, oxidative stress, and apoptosis [6, 7]. Recent studies have revealed a potential pattern of information transfer in which exosomes play a crucial role in disease development [8, 9, 10]. Exosomes, which are small vesicles with diameters that range from 40 to 100 nm, are released by a variety of cells, including neurons, microglia, and astrocytes. By carrying many molecules, including DNAs, RNAs, proteins, and metabolites, exosomes mediate intercellular communication related to many aspects of brain pathophysiology through circulation, regulate the phenotype of target cells, and facilitate communication between brain cells and between the brain and the periphery [11, 12, 13]. Studies have revealed that exosomes are promising potential diagnostic biomarkers for many neuropsychiatric disorders due to their ability to reflect pathological changes in inaccessible areas such as the brain [14, 15]. Additionally, exosomes and exosomal molecules have been demonstrated to play crucial roles in various neuropsychiatric diseases, including Alzheimer's disease, schizophrenia, depressive disorder, cerebral ischemia, and PD [16, 17]. Tau protein, which is a major hallmark of Alzheimer's disease, is spread via exosome secretion, and this spread can be reduced by exosome inhibition [18]. Of particular interest, our previous study revealed that exosomes from the blood of patients with schizophrenia transferred schizophrenia‐like behaviors to normal mice [19]. Moreover, exosomes from the blood of patients with major depressive disorder have been demonstrated to cause depressive‐like behavior in healthy mice, and exosomes from the blood of healthy volunteers alleviated depressive‐like behaviors in unpredictable mild stress‐exposed mice via miR‐139‐5p‐regulated neurogenesis [20]. Therefore, we have reason to believe that exosomes that are released by cells under different pathological or physiological conditions can not only dynamically present current information about their parent cells but also perform specific functions. Our latest study showed that exosomes from the blood of healthy volunteers attenuated motor dysfunction behaviors in PD model mice [21]. However, the functional involvement of exosomes and/or exosomal molecules in PD onset and development remains very limited. Our previous research revealed a significant decrease in adenine in both brain‐derived and blood‐derived exosomes from PD model mice [21]. Adenine, a type of purine base, serves as a fundamental unit in biosynthesis and metabolism, participating in the formation of various essential intermediates [22, 23]. Studies have demonstrated that adenine is converted into cyclic adenosine monophosphate (cAMP) in cells and tissues, thereby activating the cAMP/protein kinase A (PKA) pathway [24, 25]. The cAMP/PKA pathway is a crucial molecule in the adenine salvage synthesis pathway, and the cAMP‐PKA signaling cascade plays a central role in regulating neuronal activity and energy metabolism. Studies have confirmed that the activation of cAMP‐PKA signaling contributes to the improvement of PD [26, 27]. These findings suggest that adenine may ameliorate PD by influencing the cAMP‐PKA signaling cascade.

In this study, to elucidate the role of exosomes in the pathogenesis of PD, we transplanted exosomes from the blood of healthy rats into mice with 1‐methyl‐4‐phenyl‐1,2,3,6‐tetrahydropyridine hydrochloride (MPTP)‐induced PD and transplanted exosomes from the blood of MPTP‐treated rats into healthy mice. Then, we evaluated the motor coordination ability, pathological state, and molecular function of the mice. We further screened abnormal exosomal metabolites and manipulated the levels of one metabolite, adenine, through peripheral and intracranial injection to investigate its contribution to the development of PD.

## 2 Methods

### 2.1 Animals and MPTP Treatment

Male C57BL/6 mice (nine weeks old) and male Sprague–Dawley rats (three months old) were obtained from Vital River Laboratory (Beijing, China). The animals were housed in a temperature‐ and humidity‐controlled room under a 12 h light/dark cycle, and food and water were provided *ad libitum*. Animal procedures were approved by the Animal Care and Use Committee of the Minzu University of China.

MPTP (Macklin, Shanghai, China) was used to establish the model of PD. Following the standard procedure, the mice and rats were intraperitoneally injected with MPTP (20 mg/kg, dissolved in saline) every 2 h for a total of four doses over an 8‐h period. The control mice and rats were injected with saline using the same protocol.

### 2.2 Preparation of Exosomes and Treatments

Blood was obtained from rats via cardiac puncture. The isolation of exosomes from blood and exosome validation were performed as previously reported [28]. Briefly, exosomes were isolated from diluted blood using a qEV column and concentrated with Vivaspin (Sartorius, Gottingen, Germany). The concentrated exosomes were then resuspended in phosphate‐buffered saline (PBS) to a concentration of 600 μg/mL. Each mouse received a tail vein injection of 200 μL exosomes every 3 days, with a total of 4 times or 12 injections administered according to the experimental protocol. Thirty male SD rats were used in this study to obtain the blood‐derived exosomes for experiments, and the protocol was approved by the ethics committee.

For widely targeted metabolomics detection and quantification experiments, blood from healthy mice and MPTP‐induced mice was obtained through ocular bleeding, and brains were obtained following euthanasia of the mice using sodium pentobarbital. Exosomes were isolated from both blood and brain of the mice, with exosome validation conducted as previously reported [29]. Widely targeted metabolomics detection and quantification were conducted by METWARE [21].

### 2.3 Stereoscopic Injection Into the Substantia Nigra of Mice

Mice were anesthetized with isoflurane and immobilized in a brain stereo locator (RWD Life Science Co. Ltd., Shenzhen, China), following implantation with cannulas (RWD Life Science Co. Ltd., Shenzhen, China) in the substantia nigra (bregma: −3.3 mm, midline: 1.4 mm, DV: −4.4 mm). The mice were monitored on a heating pad until ambulatory. After two days of recovery from surgery, the mice were intracerebrally injected with 5 μL of drug or saline at a rate of 500 nL/min, and the solutions were allowed to diffuse.

### 2.4 Drugs

Adenine was obtained from MedChemExpress (Shanghai, China), dissolved in saline, and heated to facilitate complete dissolution. In the peripheral injection of adenine experiment, 20 mg/kg/day adenine was administered one hour before MPTP administration and for two weeks afterward. For the intracerebral injection of adenine, mice were intracerebrally injected with 5 μL adenine (2 mg adenine dissolved in 1 mL saline) or saline every three days for a total 12 times.

### 2.5 The Rotarod Test

The rotarod test was used to assess motor coordination ability and performed as previously reported. After 3 days of rotarod exercise training, the mice were subjected to the test before MPTP injection and after the experiment, and the fall latency of the mice was recorded. The experimental protocol was followed as previously reported. Briefly, in the training experiment, the mice were pretrained at 10 rpm for 3 days until they were able to stay on the pole for at least 90 s. In the formal test, the rotating drum accelerated from 4 rpm to 40 rpm within 5 minutes. Each trial continued until the mice could no longer stay on the pole without falling off. The latency until the mice fell from the rotating rod was recorded.

### 2.6 Immunohistochemistry

After the rotarod test, mice were euthanized with pentobarbitone and perfused with saline. The left and right hemispheres of the mice were separated and preserved using different methods for subsequent experiments. The left brains were postfixed in 4% paraformaldehyde overnight at 4°C and then dehydrated in 20% and 30% sucrose. The frozen brains were cut into 40 μm coronal sections, and immunohistochemistry staining was performed as previously reported [7]. One out of every four successive brain sections was stained. The sections were incubated overnight with rabbit anti‐tyrosine hydroxylase antibodies (TH) (1:500, CST, Boston, MA, USA) at 4°C, followed by incubation with goat anti‐rabbit antibodies (1: 400, ZSGB‐BIO, Beijing, China) for 1 h at 25°C. The sections were mounted on gelatin‐coated slides, dried, dehydrated, and coverslipped. The histological images were examined using a bright‐field microscope (Olympus, Tokyo, Japan). The number of TH‐positive neurons in each SNpc was calculated with ImageJ software (NIH, MD, USA). Subsequently, the neuron count from all SNpc region slices of each mouse was summed, and the total count was multiplied by 4 (as one out of every four successive brain sections were stained) to estimate the total number of TH‐positive neurons in the whole brain. The TH‐positive area in the striatum was calculated by ImageJ software (NIH, MD, USA).

### 2.7 Western Blotting Analysis

The SNpc and striatum were isolated and homogenized with RIPA buffer. A BCA Protein Assay Kit (Thermo Fisher, USA) was used to assess the protein concentration. Western blotting analyses were performed as previously described. A rabbit anti‐TH antibody (1:1000, BM4568, Bioster), rabbit anti‐PKA alpha/beta antibody (1:1000, 3927S, CST, USA), rabbit anti‐phospho‐PKA alpha/beta (Thr197) antibody (1:1000, 5661S, CST, USA), rabbit anti‐CD63 (1:1000, 52090S, CST, USA), rabbit anti‐HSP70 (1:1000, 4873S, CST, USA), rabbit anti‐GM130 (1:1000, 12480S, CST, USA) and mouse β‐actin antibody (1:5000) from CST, as well as a goat anti‐rabbit secondary antibody (1:10,000) and goat anti‐mouse secondary antibody (1:10,000) from CST, were used. The full unedited gel/blot for images is shown in Figure S1.

### 2.8 Quantitative Real‐Time Polymerase Chain Reaction

Total RNA was extracted from the SNpc and striatum with TRIzol reagent. cDNA was synthesized by a PrimeScript RT reagent kit and quantified by real‐time polymerase chain reaction on a LightCycler 96 (Roche, Basel, Switzerland). The relative mRNA levels were measured using β‐actin housekeeping genes as denominators for the comparison of samples. Primers were synthesized by Sangon Biotech (Shanghai) Co. Ltd. IL‐1β Forward: GTCGCTCAGGGTCACAAGAA, Reverse: CTGCTGCCTAATGTCCCCTT. IL‐6 Forward: GCTACCAAACTGGATATAATCAGGA, Reverse: CCAGGTAGCTATGGTACTCCAGAA. iNOS Forward: GTTCTCAGCCCAACAATACAAGA, Reverse: GTGGACGGGTCGATGTCAC. TNF‐α Forward: CAGGCGGTGCCTATGTCTC, Reverse: CGATCACCCCGAAGTTCAGTAG. β‐actin Forward: GGCTGTATTCCCCTCCATCG, Reverse: CCAGTTGGTAACAATGCCATGT.

### 2.9 Oxidative Stress Marker Level/Activity Measurement

The malondialdehyde (MDA) level and total antioxidant capacity (T‐AOC) activity in serum were measured by a colorimetric assay kit (Jiancheng Bioengineering Institute, China) according to the manufacturer's instructions.

### 2.10 Enzyme‐Linked Immunosorbent Assay (ELISA)

The cyclic adenosine monophosphate (cAMP) levels in the SNpc and striatum were measured using ELISA kits (Elabscience Biotechnology Co. Ltd., China) following the manufacturer's instructions. The protein concentrations of the supernatants were measured using a BCA Protein Assay Kit, and the cAMP levels are expressed as pmol/mg protein.

### 2.11 Statistical Analysis

Statistical analyses were performed using GraphPad Prism 9. The Shapiro–Wilk method was used to test the normal distribution of all data. One‐way ANOVA with Tukey's post hoc test or Student's unpaired *t* test was used to analyze the data. The data are reported as the mean ± standard deviation (S.D.). Differences with a *p* value < 0.05 were considered statistically significant.

## 3 Results

### 3.1 Characterizations of Exosomes From the Blood of Rats and Mice

**FIGURE 1:**
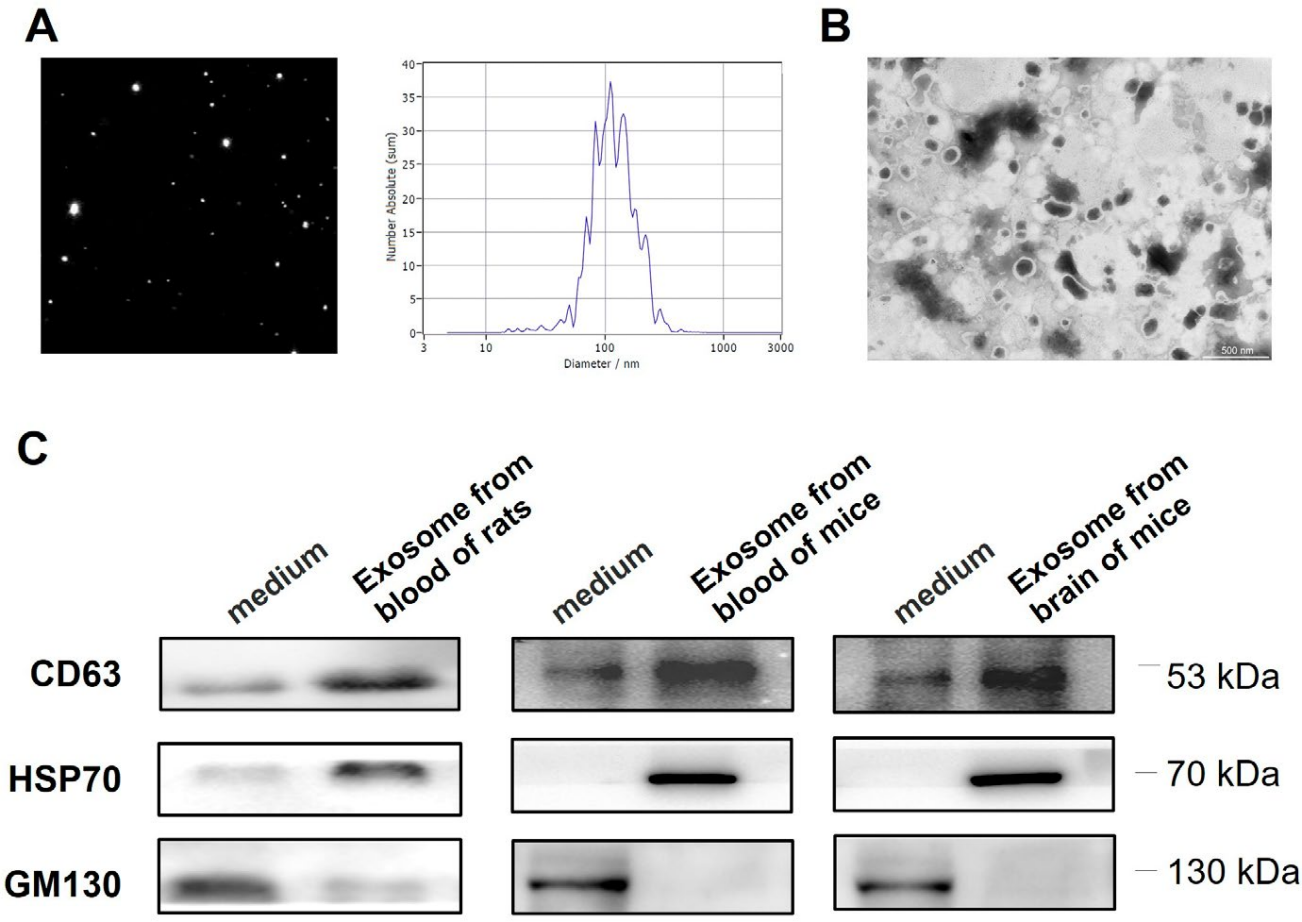
Characterization of exosomes from rats and mice. (A) Nanoparticle tracking analysis (NTA) was performed to determine the size distribution of exosomes derived from the blood of rats. (B) The spheroid morphology and size of exosomes derived from the blood of rats were analyzed by TEM. (C) The protein levels of CD63, HSP70, and GM130 in exosomes derived from the blood of rats and the brain of mice were assessed using western blotting.

Exosomes from the blood of rats isolated using size exclusion columns (qEV) were identified. Nanoparticle tracking analysis (NTA) revealed that the size distribution of exosomes ranged from 90 to 120 nm (Figure 1A). Exosomes appeared to be typical lipid bilayer membrane‐encapsulated nanoparticles according to transmission electron microscopy (TEM) (Figure 1B). According to the minimal experimental requirements for exosome identification stipulated by the International Society for Extracellular Vesicles (MISEV2018) [30], exosomes should be characterized by at least three markers, including one transmembrane protein, one cytosolic protein, and one negative protein marker. We utilized Western blot analyses to identify the transmembrane protein CD63, the cytosolic protein HSP70, and the negative protein marker Golgi protein GM130. The results demonstrated that the extracted exosomes from blood of rats, blood, and brain of mice were positive for CD63 and HSP70, and negative for GM130, aligning with the characteristics of exosomes (Figure 1C). These results demonstrated the reliability of isolated exosomes.

### 3.2 Healthy Exosomes From the Blood of Rats Attenuated Motor Dysfunction and Pathological Manifestations in MPTP‐Treated Mice

**FIGURE 2:**
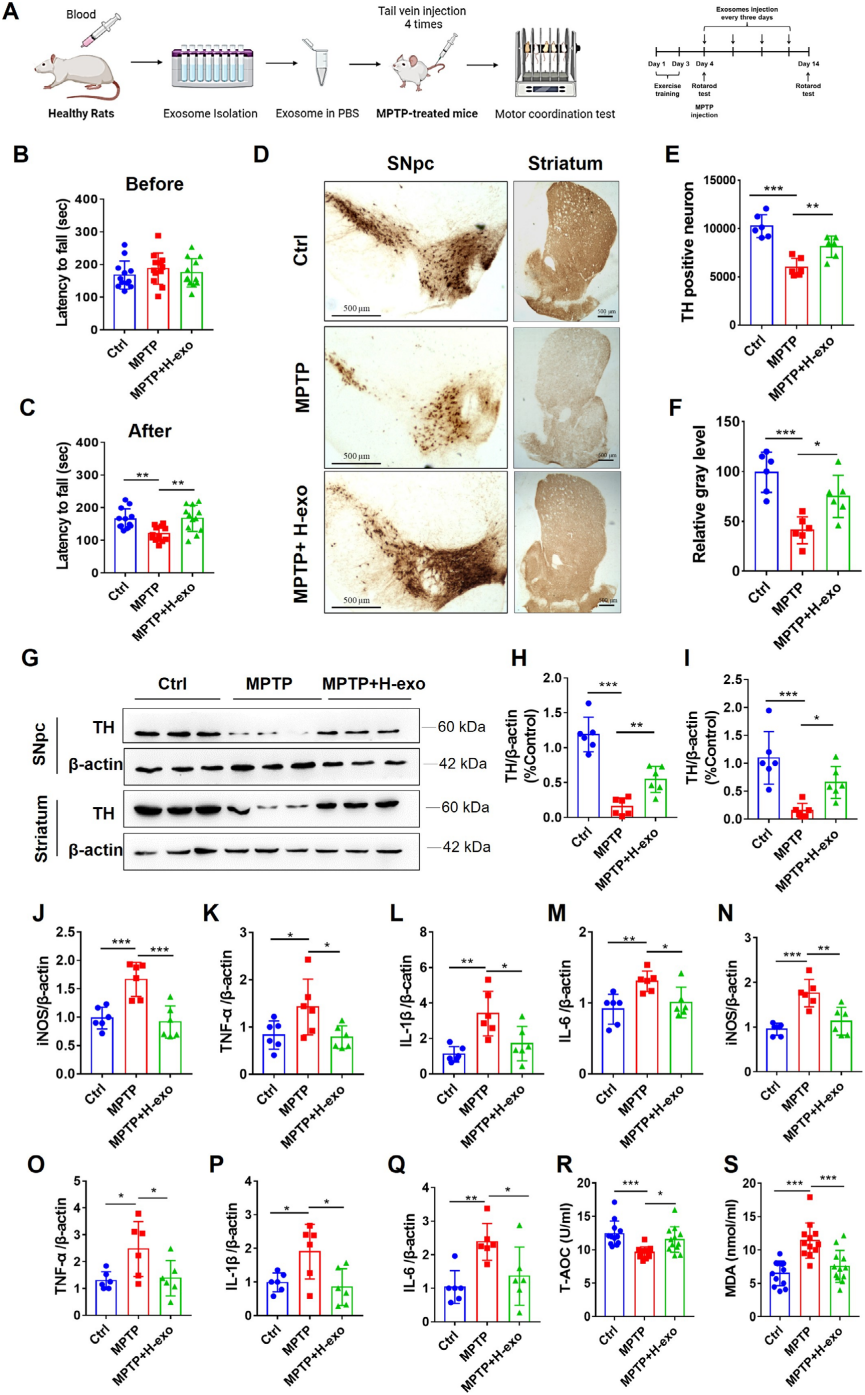
Healthy exosomes from the blood of rats’ attenuated motor dysfunction and pathological injury in MPTP‐treated mice. (A) Experimental design for the treatment of MPTP‐treated mice with healthy exosomes. (B, C) Latency to fall in the rotarod test of mice before MPTP injection (B) and after MPTP injection (C), *n* = 12. (D) Representative immunohistochemical staining of TH‐positive neurons in the SNpc and striatum of mice. (E) Quantification of the number of TH‐positive neurons in the SNpc, *n* = 6. (F) Relative gray levels of TH‐positive immunohistochemical staining in the striatum, *n* = 6. (G) Representative immunoblots showing TH expression in the SNpc and striatum. (H, I) Quantification of TH expression in the SNpc (H) and striatum (I), *n* = 6. (J–Q) qRT–PCR analyses of iNOS (J), TNF‐α (K), L‐1β (L), and IL‐6 (M) expression in the SNpc and iNOS (N), TNF‐α (O), L‐1β (P), and IL‐6 (Q) expression in the striatum, *n* = 6. (R) T‐AOC levels in the serum of mice, *n* = 12. (S) MDA levels in the serum of mice, *n* = 12. All values are means ± S.D., * *p* < 0.05, ** *p* < 0.01, and *** *p* < 0.001.

We first peripherally injected exosomes from the blood of healthy rats into MPTP‐treated mice to assess the functional involvement of blood‐derived exosomes in PD (Figure 2A). The motor coordination of the mice was measured by the fall latency of the rotarod test. All the groups of mice presented equal motor coordination abilities before MPTP injection, as manifested by the fall latency of the mice in the rotarod test (Figure 2B *F*_(2, 33)_ = 0.5767, *p* > 0.05). MPTP treatment significantly reduced the fall latency of mice, and healthy exosome treatment significantly attenuated the MPTP‐induced decrease in fall latency (Figure 2C *F*_(2, 33)_ = 8.421, *p* < 0.01; *p* < 0.01 Ctrl vs. MPTP and MPTP vs. MPTP+H‐exo).

Loss of dopamine neurons is one of the major features of PD, and tyrosine hydroxylase (TH) is the typical marker of dopaminergic neurons. Immunohistochemical staining for TH indicated that the number of TH‐positive neurons was significantly reduced in the SNpc of MPTP‐treated mice (Figure 2D,E *F*_(2, 15)_ = 23.39, *p <* 0.001; *p* < 0.001 Ctrl vs. MPTP). However, healthy exosome treatment greatly reversed this effect (Figure 2D,E *F*_(2, 15)_ =23.39, *p <* 0.001; *p* < 0.01 MPTP vs. MPTP+H‐exo). Similar results were also observed in the striatum (Figure 2D,F *F*_(2, 15)_ = 14.77, *p <* 0.05; *p* < 0.001 Ctrl vs. MPTP; *p* < 0.05 MPTP vs. MPTP+H‐exo). Moreover, western blotting analysis showed that TH levels were significantly decreased in the SNpc and striatum of MPTP‐treated mice but increased in the SNpc and striatum of mice treated with healthy exosomes (Figure 2G–I, SNpc: *F*_(2, 15)_ = 43.84, *p <* 0.01, *p* < 0.001 Ctrl vs. MPTP; *p* < 0.01 MPTP vs. MPTP+H‐exo; striatum: *F*_(2, 15)_ = 12.53, *p <* 0.05, *p* < 0.001 Ctrl vs. MPTP; *p* < 0.05 MPTP vs. MPTP+H‐exo). Together, these findings revealed that healthy exosome treatment alleviates motor dysfunction and dopamine neuron damage in PD model mice.

We next explored inflammation in response to exosome treatment. The mRNA levels of iNOS, TNF‐α, IL‐1β, and IL‐6 were significantly increased in the SNpc (Figure 2J–M, iNOS: *F*_(2, 15)_ = 14.93, *p <* 0.001, *p <* 0.001 Ctrl vs. MPTP; TNF‐α: *F*_(2, 15)_ = 4.602, *p <* 0.05, *p <* 0.05 Ctrl vs. MPTP; IL‐1β: *F*_(2, 15)_ = 9.358, *p <* 0.01, *p <* 0.05 Ctrl vs. MPTP; IL‐6: *F*_(2, 15)_ = 6.772, *p <* 0.01, *p <* 0.05 Ctrl vs. MPTP) and striatum (Figure 2N–Q, iNOS: *F*_(2, 15)_ = 15.6, *p <* 0.001, *p <* 0.001 Ctrl vs. MPTP; TNF‐α: *F*_(2, 15)_ = 4.862, *p <* 0.05, *p <* 0.05 Ctrl vs. MPTP; IL‐1β: *F*_(2, 15)_ = 5.711, *p <* 0.05, *p <* 0.05 Ctrl vs. MPTP; IL‐6: *F*_(2, 15)_ = 6.883, *p <* 0.01, *p <* 0.001 Ctrl vs. MPTP) of MPTP‐treated mice, and these increases were reversed by healthy exosome treatment (Figure 2J–Q, iNOS in SNpc: *p <* 0.001; TNF‐α in SNpc: *p <* 0.05; IL‐1β in SNpc: *p <* 0.01; IL‐6 in SNpc: *p <* 0.01; iNOS in striatum: *p <* 0.01; TNF‐α in striatum: *p <* 0.05; IL‐1β in striatum: *p <* 0.05; IL‐6 in striatum: *p <* 0.05; MPTP vs. MPTP+H‐exo).

The serum total antioxidant capacity (T‐AOC) and malondialdehyde (MDA) levels were examined to assess oxidative damage and antioxidant ability. The results revealed that the T‐AOC level was significantly reduced in MPTP‐treated mice but increased in response to healthy exosome treatment (Figure 2R *F*_(2, 33)_ = 9.392, *p <* 0.001, *p <* 0.001 Ctrl vs. MPTP, *p <* 0.05 MPTP vs. MPTP+H‐exo). Similarly, the MDA level was significantly increased by MPTP, but this effect was reversed by healthy exosomes (Figure 2S *F*_(2, 33)_ = 15.16, *p <* 0.001, *p <* 0.001 Ctrl vs. MPTP and MPTP vs. MPTP+H‐exo). These findings suggested that healthy exosome treatment attenuates neuroinflammation and oxidative damage in PD model mice.

### 3.3 Exosomes From MPTP‐Treated Rats Caused PD‐Like Motor Dysfunction and Pathology in mice

**FIGURE 3:**
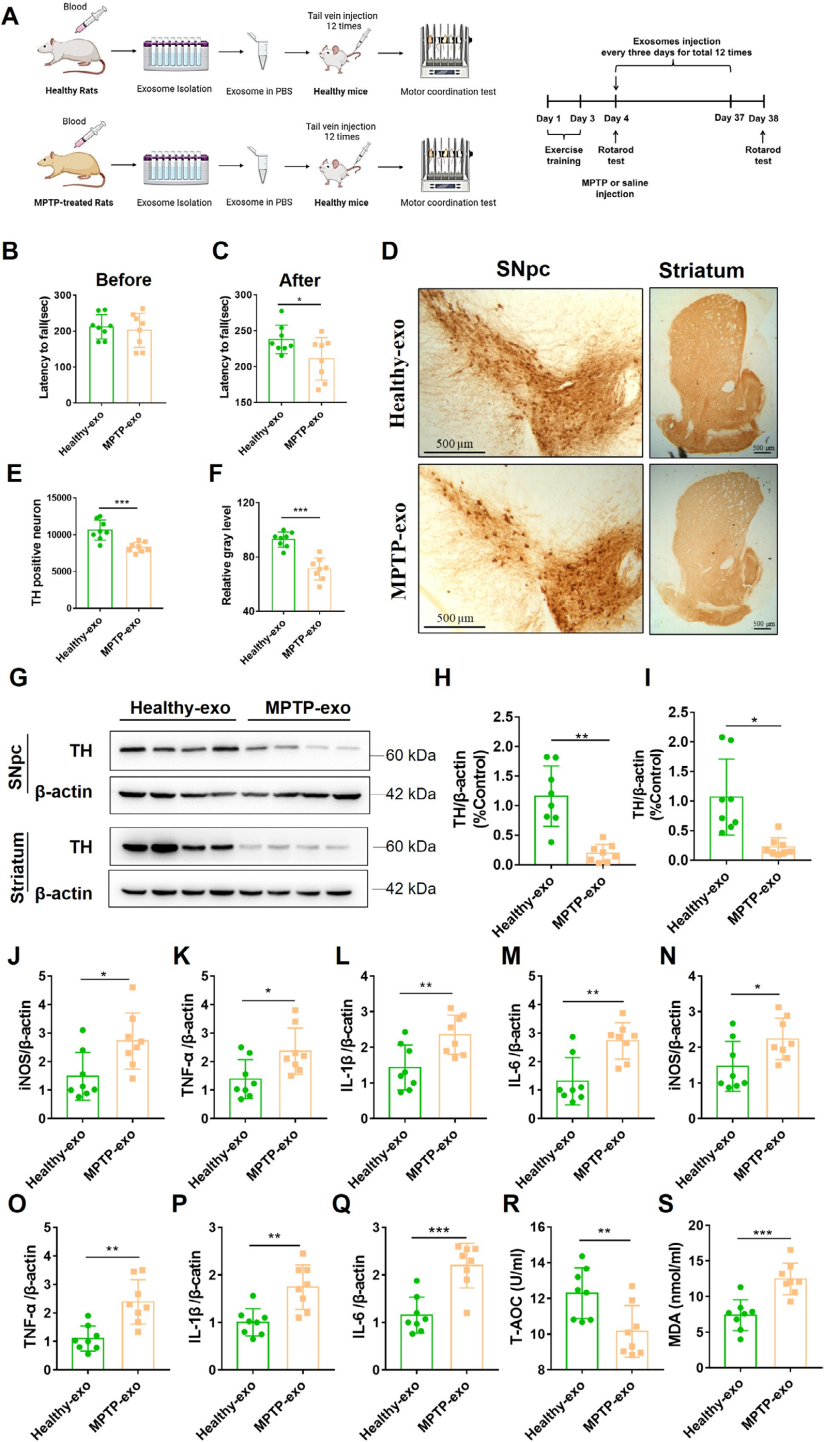
Exosomes from MPTP‐treated rats caused PD‐like motor dysfunction and pathology in mice. (A) Experimental design of the treatment of mice with healthy exosomes and MPTP exosomes. (B, C) Latency to fall in the rotarod test of mice before MPTP injection (B) and after MPTP injection (C), *n* = 8. (D) Representative immunohistochemical staining of TH‐positive neurons in the SNpc and striatum of mice. (E) Quantification of the number of TH‐positive neurons in the SNpc, *n* = 8. (F) Relative gray levels of TH‐positive immunohistochemical staining in the striatum, *n* = 8. (G) Representative immunoblots showing TH expression in the SNpc and striatum. (H, I) Quantification of TH expression in the SNpc (H) and striatum (I), *n* = 8. (J–Q) qRT–PCR analyses of iNOS (J), TNF‐α (K), L‐1β (L), and IL‐6 (M) expression in the SNpc and iNOS (N), TNF‐α (O), L‐1β (P), and IL‐6 (Q) expression in the striatum, *n* = 8. (R) T‐AOC levels in the serum of mice, *n* = 8. (S) MDA levels in the serum of mice, *n* = 8. All values are means ± S.D., * *p* < 0.05, ** *p* < 0.01, and *** *p* < 0.001.

After confirming the neuroprotective effects of healthy exosomes, we further assessed the functional involvement of blood‐derived exosomes in PD. We isolated exosomes from the blood of rats that were treated with MPTP. These exosomes were named PD exosomes and injected into healthy mice via the tail vein (Figure 3A).

The rotarod test results showed that accumulated PD‐exosome injection caused a reduction in the fall latency of the mice, indicating that the motor coordination of the mice was impaired (Figure 3B,C, before rotarod test *t =* 0.4784, *df =* 14, *p* > 0.05; after rotarod test *t =* 2.146, *df =* 14, *p* < 0.05). Moreover, immunohistochemical staining for TH demonstrated a significant decrease in TH‐positive neurons in the SNpc (Figure 3E, *t =* 4.347, *df =* 14, *p* < 0.001) and striatum (Figure 3F, *t =* 6.213, *df =* 14, *p* < 0.001) of MPTP‐exosome‐treated mice. Additionally, the TH protein levels in the SNpc (Figure 3G,H, *t =* 5.118, *df =* 14, *p* < 0.001) and striatum (Figure 3G,I, *t =* 3.612, *df =* 14, *p* < 0.01) yielded the same results, namely, that PD‐exosomes contributed to the decrease in TH protein expression. These findings suggested that PD‐exosomes triggered motor dysfunction and dopamine neuron damage in healthy mice.

To assess whether PD‐exosomes induce an inflammatory reaction, the mRNA levels of inflammatory cytokines in the SNpc and striatum were measured. The results showed that PD‐exosome treatment increased the levels of iNOS (Figure 3J, *t =* 2.71, *df =* 14, *p* < 0.05), TNF‐α (Figure 3K, *t =* 2.598 *df =* 14, *p* < 0.05), IL‐1β (Figure 3L, *t =* 3.108, *df =* 14, *p* < 0.01), and IL‐6 (Figure 3M, *t =* 3.838, *df =* 14, *p* < 0.01) in the SNpc and iNOS (Figure 3N, *t =* 2.388, *df =* 14, *p* < 0.05), TNF‐α (Figure 3O, *t =* 4.076, *df =* 14, *p* < 0.01), IL‐1β (Figure 3P, *t =* 3.823, *df =* 14, *p* < 0.01), and IL‐6 (Figure 3Q, *t =* 3.4884, *df =* 14, *p* < 0.001) in the striatum. Similarly, PD‐exosome treatment also caused oxidative damage, as manifested by reduced T‐AOC levels (Figure 3R, *t =* 2.983, *df =* 14, *p* < 0.01) and increased MDA levels (Figure 3S, *t =* 2.983, *t =* 4.64, *df =* 14, *p* < 0.001).

### 3.4 Peripherally Injected Adenine Alleviated PD‐Like Behaviors and Pathology in Mice

**FIGURE 4:**
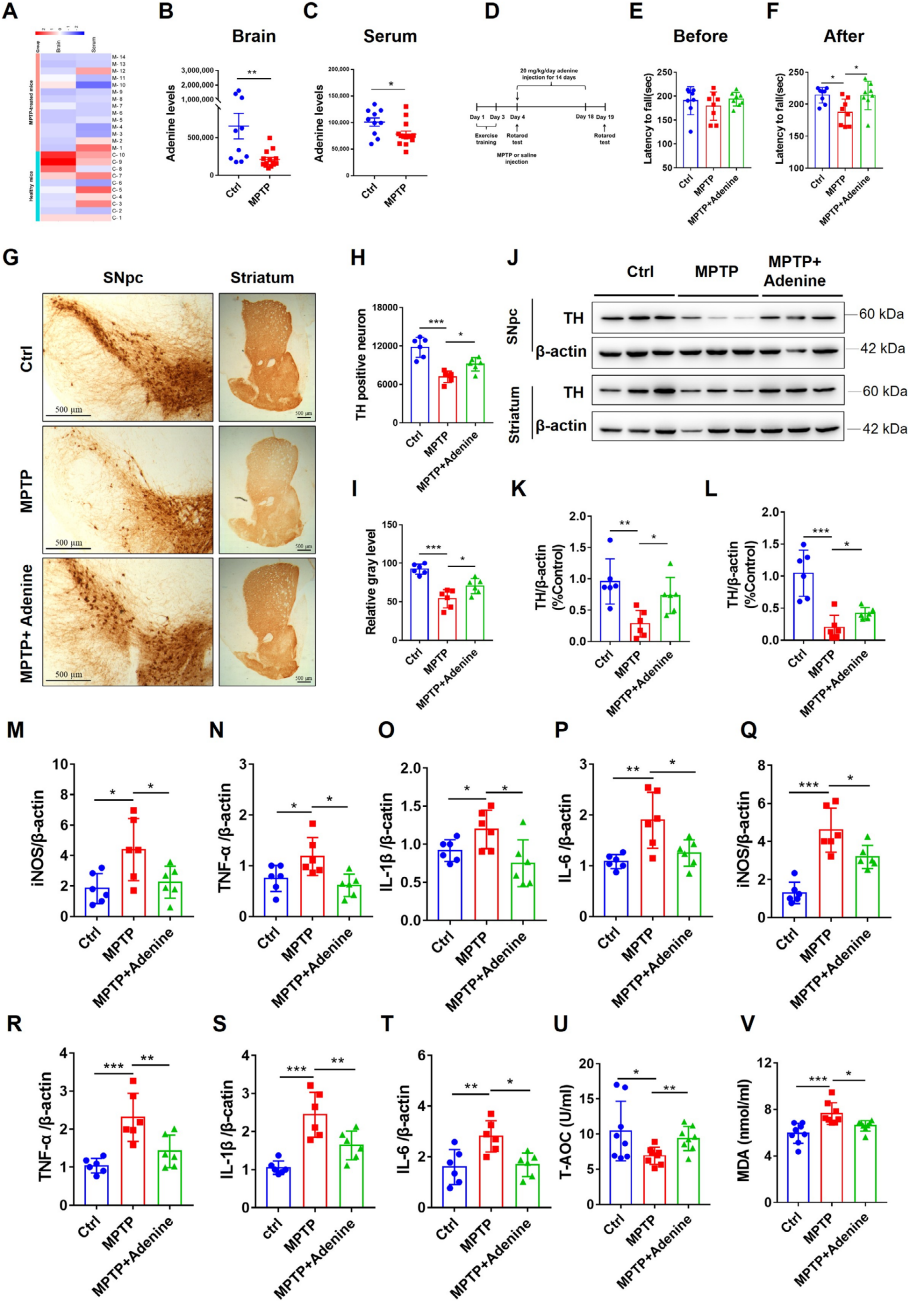
Peripheral injection of adenine alleviated PD‐like behaviors and pathology in mice. (A) Heatmap of exosomal adenine levels in the brain and serum of mice tested by widely targeted metabolomics, *n* = 10–14. (B, C) Adenine levels in the brain (B) and serum (C) of mice as determined by widely targeted metabolomics, *n* = 10–14. (D) Experimental design of the intraperitoneal injection of adenine to treat mice. (E, F) Latency to fall in the rotarod test for mice before MPTP injection (E) and after MPTP injection (F), *n* = 8. (G) Representative immunohistochemical staining of TH‐positive neurons in the SNpc and striatum of mice. (H) Quantification of the number of TH‐positive neurons in the SNpc, *n* = 6. (I) Relative gray levels of TH‐positive immunohistochemical staining in the striatum. (J) Representative immunoblots showing TH expression in the SNpc and striatum, *n* = 6. (K, L) Quantification of TH expression in the SNpc (K) and striatum (L), *n* = 6. (M–T) qRT–PCR analyses of iNOS (M), TNF‐α (N), L‐1β (O), and IL‐6 (P) expression in the SNpc and iNOS (Q), TNF‐α (R), L‐1β (S), and IL‐6 (T) expression in the striatum, *n* = 6. (U) T‐AOC levels in the serum of mice, *n* = 6. (V) MDA levels in the serum of mice, *n* = 6. All values are means ± S.D., * *p* < 0.05, ** *p* < 0.01, and *** *p* < 0.001.

The aforementioned results confirmed that healthy exosomes have therapeutic effects in PD, while PD exosomes have pathological effects. We next explored the mechanisms underlying exosome involvement in PD pathology. In our previous study [21], a widely targeted metabolomics analysis revealed that there were several differences in the metabolites encapsulated in exosomes from the serum and brain of healthy mice and MPTP‐treated mice. Adenine was significantly reduced in the exosomes from the brain (Figure 4A,B, *t =* 3.01, *df =* 22, *p* < 0.01) and serum (Figure 4A,C, *t =* 2.34, *df =* 22, *p* < 0.05) of MPTP‐treated mice.

Furthermore, to assess the functional involvement of adenine in PD, we intraperitoneally injected adenine into normal mice and evaluated their behaviors and pathology (Figure 4D). The rotarod test results showed that adenine significantly alleviated the MPTP‐induced decrease in fall latency (Figure 4E,F, before rotarod test, *F*_(2, 21)_ = 0.7209, *p* > 0.05; after rotarod test, *F*_(2, 21)_ = 5.3, *p* < 0.05, *p* < 0.05 Ctrl vs. MPTP and MPTP vs. MPTP+Adenine).

Then, immunohistochemical staining for TH indicated that adenine significantly increased the number of TH‐positive neurons, and this number was reduced in the SNpc of MPTP‐treated mice (Figure 4G,H *F*_(2, 15)_ = 22.41, *p* < 0.001, *p* < 0.001 Ctrl vs. MPTP, *p* < 0.05 MPTP vs. MPTP+ Adenine). Similar results were also demonstrated in the striatum of mice (Figure 4G,I *F*_(2, 15)_ = 24.16, *p* < 0.001, *p* < 0.001 Ctrl vs. MPTP, *p* < 0.05 MPTP vs. MPTP+ Adenine). Additionally, western blotting analysis showed that TH levels were significantly increased in the SNpc (Figure 4J,K *F*_(2, 15)_ = 8.197, *p* < 0.001, *p* < 0.01 Ctrl vs. MPTP, *p* < 0.05 MPTP vs. MPTP+ Adenine) and striatum (Figure 4J,L *F*_(2, 15)_ = 19.92, *p* < 0.001, *p* < 0.001 Ctrl vs. MPTP, *p* < 0.05 MPTP vs. MPTP+ Adenine) of adenine‐treated mice compared with MPTP‐treated mice.

The mRNA levels of inflammatory cytokines showed that MPTP increased the levels of iNOS (Figure 4M *F*_(2, 15)_ = 5.427, *p* < 0.05, *p* < 0.05 Ctrl vs. MPTP), TNF‐α (Figure 4N *F*_(2, 15)_ =6.24, *p* < 0.05, *p* < 0.05 Ctrl vs. MPTP), IL‐1β (Figure 4O *F*_(2, 15)_ = 5.06, *p* < 0.05, *p* < 0.05 Ctrl vs. MPTP), and IL‐6 (Figure 4P *F*_(2, 15)_ = 8.526, *p* < 0.01, *p* < 0.01 Ctrl vs. MPTP) in the SNpc and iNOS (Figure 4Q *F*_(2, 15)_ = 24.47, *p* < 0.001, *p* < 0.001 Ctrl vs. MPTP), TNF‐α (Figure 4R *F*_(2, 15)_ = 12.31, *p* < 0.001, *p* < 0.001 Ctrl vs. MPTP), IL‐1β (Figure 4S *F*_(2, 15)_ = 16.78, *p* < 0.001, *p* < 0.001 Ctrl vs. MPTP), and IL‐6 (Figure 4T *F*_(2, 15)_ = 7.65, *p* < 0.01, *p* < 0.01 Ctrl vs. MPTP) in the striatum, while adenine treatment reversed these changes (Figure 4M–T, all *p <* 0.05, MPTP vs. MPTP+ Adenine). We next assessed the T‐AOC levels and MDA levels in the serum of mice. There was a significant increase in the T‐AOC levels (Figure 4U *F*_(2, 21)_ = 3.493, *p* < 0.05, *p* < 0.05 Ctrl vs. MPTP, *p* < 0.01 MPTP vs. MPTP+ Adenine) and a significant reduction in the MDA levels (Figure 4S *F*_(2, 21)_ = 9.584, *p* < 0.01, *p* < 0.001 Ctrl vs. MPTP, *p* < 0.05 MPTP vs. MPTP+ Adenine) in adenine‐treated mice compared with those in MPTP‐treated mice.

### 3.5 Adenine Intracerebral Injection Alleviated PD‐Like Behaviors and Pathology in Mice

**FIGURE 5:**
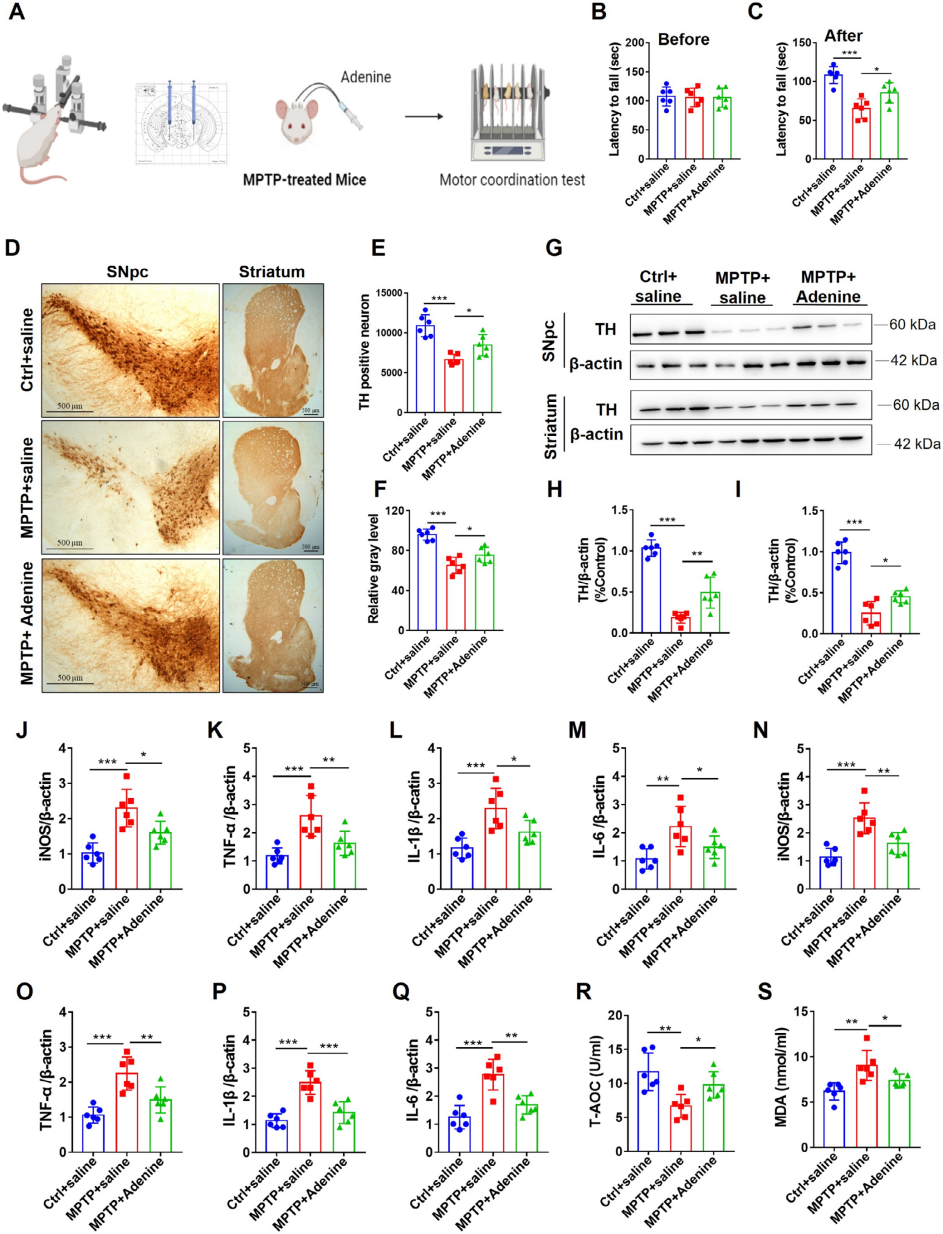
Intracerebral injection of adenine alleviated PD‐like behaviors and pathology in mice. (A) Experimental design of the intracerebral injection of adenine to treat mice. (B, C) Latency to fall in the rotarod test for mice before MPTP injection (B) and after MPTP injection (C). (D) Representative immunohistochemical staining of TH‐positive neurons in the SNpc and striatum of mice. (E) Quantification of the number of TH‐positive neurons in the SNpc. (F) Relative gray levels of TH‐positive immunohistochemical staining in the striatum. (G) Representative immunoblots showing TH expression in the SNpc and striatum. (H, I) Quantification of TH expression in the SNpc (H) and striatum (I). (J–Q) qRT–PCR analyses of iNOS (J), TNF‐α (K), L‐1β (L), and IL‐6 (M) expression in the SNpc and iNOS (N), TNF‐α (O), L‐1β (P), and IL‐6 (Q) expression in the striatum. (R) T‐AOC levels in the serum of mice. (S) MDA levels in the serum of mice. All values are means ± S.D., *n* = 6, * *p* < 0.05, ** *p* < 0.01, and *** *p* < 0.001.

We further injected adenine into the substantia nigra using stereotaxic surgery to exclude the possible effects of peripheral injection (Figure 5A). We observed that MPTP‐treated mice that were treated with adenine showed better motor coordination in the rotarod test than MPTP‐treated mice that were treated with saline (Figure 5B,C, before rotarod test, *F*_(2, 15)_ = 0.02608, *p* > 0.05; after rotarod test, *F*_(2, 15)_ = 18.71, *p* < 0.001, *p* < 0.001 Ctrl vs. MPTP, *p* < 0.05 MPTP vs. MPTP+ Adenine). Next, immunohistochemistry showed that adenine treatment mitigated MPTP‐induced dopamine neuron damage in both the SNpc (Figure 5D,E *F*_(2, 15)_ = 19.82, *p* < 0.001, *p* < 0.001 Ctrl vs. MPTP, *p* < 0.05 MPTP vs. MPTP+ Adenine) and striatum (Figure 5D,F *F*_(2, 15)_ = 28.42, *p* < 0.001, *p* < 0.001 Ctrl vs. MPTP, *p* < 0.05 MPTP vs. MPTP+ Adenine) of mice. The western blotting assay showed similar results in both the SNpc (Figure 5G,H *F*_(2, 15)_ = 66.42, *p* < 0.001, *p* < 0.001 Ctrl vs. MPTP, *p* < 0.01 MPTP vs. MPTP+ Adenine)and striatum (Figure 5G,I *F*_(2, 15)_ = 59.77, *p* < 0.001, *p* < 0.001 Ctrl vs. MPTP, *p* < 0.05 MPTP vs. MPTP+ Adenine) of mice. Subsequently, we assessed inflammation and oxidative stress in response to adenine. The results revealed that adenine inhibited the increase in iNOS (Figure 5J *F*_(2, 15)_ = 15.57, *p* < 0.001, *p* < 0.001 Ctrl vs. MPTP, *p* < 0.05 MPTP vs. MPTP+ Adenine), TNF‐α (Figure 5H *F*_(2, 15)_ = 12.07, *p* < 0.001, *p* < 0.001 Ctrl vs. MPTP, *p* < 0.01 MPTP vs. MPTP+ Adenine), IL‐1β (Figure 5L *F*_(2, 15)_ = 10.93, *p* < 0.001, *p* < 0.001 Ctrl vs. MPTP, *p* < 0.05 MPTP vs. MPTP+ Adenine), and IL‐6 (Figure 5M *F*_(2, 15)_ = 7.604, *p* < 0.01, *p* < 0.01 Ctrl vs. MPTP, *p* < 0.05 MPTP vs. MPTP+ Adenine) in the SNpc and iNOS (Figure 5N *F*_(2, 15)_ = 16.09, *p* < 0.001, *p* < 0.001 Ctrl vs. MPTP, *p* < 0.01 MPTP vs. MPTP+ Adenine), TNF‐α (Figure 5O *F*_(2, 15)_ = 15.67, *p* < 0.001, *p* < 0.001 Ctrl vs. MPTP, *p* < 0.01, MPTP vs. MPTP+ Adenine), IL‐1β (Figure 5P *F*_(2, 15)_ = 23.82, *p* < 0.001, *p* < 0.001 Ctrl vs. MPTP, *p* < 0.001 MPTP vs. MPTP+ Adenine), and IL‐6 (Figure 5Q *F*_(2, 15)_ = 19.04, *p* < 0.001, *p* < 0.001 Ctrl vs. MPTP, *p* < 0.01 MPTP vs. MPTP+ Adenine) in the striatum of MPTP‐treated mice. Moreover, adenine also suppressed oxidative damage, as manifested by increased T‐AOC levels (Figure 5R *F*_(2, 15)_ = 8.123, *p* < 0.01, *p* < 0.01 Ctrl vs. MPTP, *p* < 0.05 MPTP vs. MPTP+ Adenine) and decreased MDA levels (Figure 5S *F*_(2, 15)_ = 8.898, *p* < 0.01, *p* < 0.01 Ctrl vs. MPTP, *p* < 0.01 MPTP vs. MPTP+ Adenine).

### 3.6 Adenine Activates cAMP/PKA Signaling via the Purine Synthesis Remedy Pathway

**FIGURE 6:**
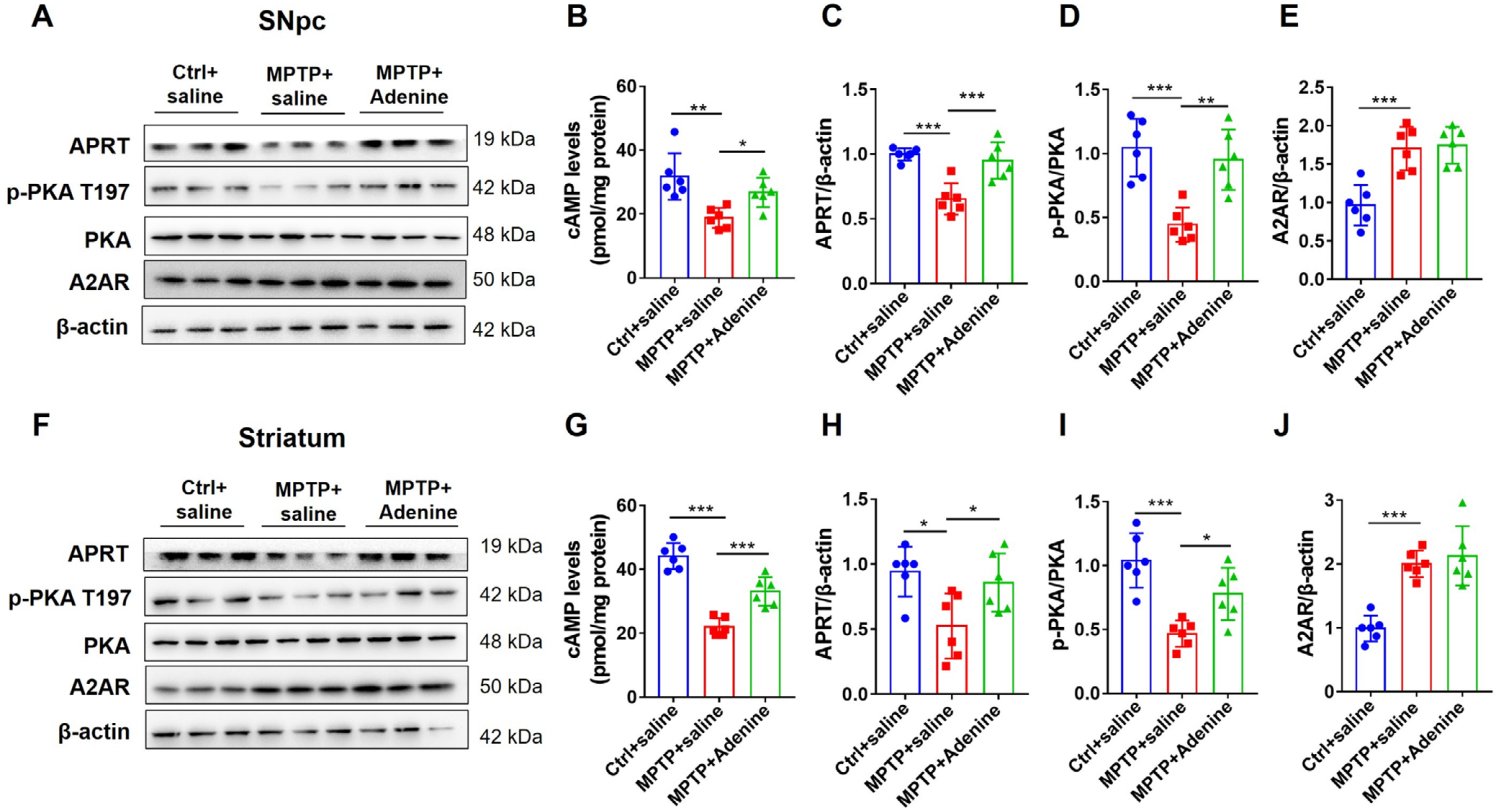
Adenine activates cAMP/PKA signaling via the purine synthesis remedy pathway. (A and F) Representative immunoblots showing APRT, p‐PKA T197, PKA, A2AR, and β‐actin expression in the SNpc (A) and striatum (F). (B and G) cAMP levels in the SNpc (B) and striatum (G) by ELISA. (C–E) Quantification of APRT (C), p‐PKA T197/PKA (D), and A2AR (E) expression in the SNpc. (H–J) Quantification of APRT (H), p‐PKA T197/PKA (I) and A2AR (J) expression in the striatum. All values are means ± S.D., *n* = 6, * *p* < 0.05, ** *p* < 0.01, and *** *p* < 0.001.

Adenine phosphoribosyl transferase (APRT) is a key enzyme in the purine synthesis remedy pathway whose substrate is adenine and product is AMP. The western blotting results revealed that APRT protein levels were significantly decreased in the SNpc (Figure 6A,C *F*_(2, 15)_ = 17.03, *p* < 0.001, *p* < 0.001 Ctrl vs. MPTP, *p* < 0.001 MPTP vs. MPTP+ Adenine) and striatum (Figure 6F,H *F*_(2, 15)_ = 5.861, *p* < 0.05, *p* < 0.05 Ctrl vs. MPTP, *p* < 0.05 MPTP vs. MPTP+ Adenine) of MPTP‐treated mice but increased in mice treated with adenine. Moreover, adenine has been shown to activate cAMP/protein kinase A (PKA) signaling by increasing cAMP levels and PKA phosphorylation, which is manifested by increased cAMP (Figure 6B,G, SNpc: *F*_(2, 15)_ = 9.147, *p* < 0.01, *p* < 0.01 Ctrl vs. MPTP, *p* < 0.05 MPTP vs. MPTP+ Adenine; striatum: *F*_(2, 15)_ = 50.08, *p* < 0.001, *p* < 0.001 Ctrl vs. MPTP, *p* < 0.001 MPTP vs. MPTP+ Adenine) and p‐PKA/PKA levels (Figure 6A,D,F,I, SNpc: *F*_(2, 15)_ = 15.1, *p* < 0.001, *p* < 0.001 Ctrl vs. MPTP, *p* < 0.01 MPTP vs. MPTP+ Adenine; striatum: *F*_(2, 15)_ = 15.1, *p* < 0.001, *p* < 0.001 Ctrl vs. MPTP, *p* < 0.05 MPTP vs. MPTP+ Adenine). Adenosine A2A receptor (A2AR), a target of adenosine, plays an important role in dopaminergic neural circuits. To investigate whether adenine activates the adenosine signaling mechanism, A2AR protein expression levels were measured by western blotting analysis. The results showed that adenine treatment had no significant effect on A2AR protein expression in the SNpc (Figure 6A,E *F*_(2, 15)_ = 16.8, *p* < 0.001, *p* < 0.001 Ctrl vs. MPTP, *p* > 0.05 MPTP vs. MPTP+ Adenine) and striatum (Figure 6F,J *F*_(2, 15)_ = 23.54, *p* < 0.001, *p* < 0.001 Ctrl vs. MPTP, *p* > 0.05 MPTP vs. MPTP+ Adenine) of MPTP‐treated mice. Together, these findings revealed that adenine activates cAMP/PKA signaling via the purine synthesis remedy pathway rather than via adenosine signaling.

## 4 Discussion

Exosomes have been recognized as promising diagnostic biomarkers, therapeutic agents, and drug delivery carriers for multiple diseases and have been widely investigated in various studies [31, 32, 33]. However, the function and mechanism of exosomes in PD have not been revealed. In the present study, we demonstrated that exosomes derived from the blood of healthy rats have therapeutic effects on motor dysfunction and pathology in PD mice; conversely, exosomes released by PD model rats cause PD‐like pathological features in healthy mice. Moreover, adenine, which is enriched in healthy exosomes, plays a key role in PD pathology by activating cAMP/PKA signaling via the purine synthesis remedy pathway.

PD is the most common motor dysfunction and the second most prevalent neurodegenerative disease. It is incurable, and it has an unknown etiology in most cases. Several studies have attempted to explore the treatment and pathogenesis of PD by focusing on endogenous molecules, such as exosomes originating from mesenchymal stromal cells, microglia, and astrocytes [34, 35, 36]. Exosomes, which are nanovesicles released by various types of cells, play a crucial role in intercellular communication and have great potential as therapeutic agents [37, 38]. Numerous studies have demonstrated that exosomes from various cells or tissues exert palliative or therapeutic effects on nervous system diseases. Research has revealed that exosomes from the blood of healthy volunteers alleviate depressive‐like behavior in a mouse model of major depressive disorder [20]. Exosomes released by oxygen–glucose deprivation/reoxygenation‐stimulated astrocytes reduce neuronal death and promote neuronal autophagy through the release of Nampt, which targets AMPK/mTOR signaling pathways [39]. Small extracellular vesicles secreted by nigrostriatal astrocytes have been found to rescue cell death and preserve mitochondrial function in PD [40]. Our previous study also noted that exosomes derived from healthy volunteers ameliorate impaired motor coordination, dopaminergic neuron loss, and apoptosis in a mouse model of PD. In the current study, we revealed that exosomes from the blood of healthy rats ameliorated motor dysfunction, reversed the loss of dopaminergic neurons, and restored the homeostasis of oxidative stress and neuroinflammation in PD model mice. All these findings imply that exosomes, as a new therapeutic agent, perform a beneficial function in alleviating PD pathology. It is worth noting that exosomes may also be a novel factor in the pathogenesis of PD based on these inferences. Exosome secretion is one of the most widely studied pathways for the secretion of pathologic molecules [41, 42]. A study has shown that PD patient‐derived extracellular vesicles exacerbate pathology and neuronal loss in Prnp‐SNCA^A53T^ transgenic PD mice [43]. In our present study, we revealed the pathological effects of blood‐derived exosomes from PD model rats on healthy mice. Intravenous treatment of mice with PD‐exosomes recapitulated the behavioral phenotypes and pathology of PD, including motor dysfunction, dopaminergic neuron loss, oxidative injury, and neuroinflammation. The presence of PD‐like pathological features in recipient animals suggests that exosomes serve as carriers and transmitters of pathological information to the recipients, while the latter accept these foreign signals and transmit their contents accordingly. Our findings further confirm that exosomes transmit pathological information related to PD and confirm that the dual effect of exosome production is dependent on the origin of exosomes.

Exosomes seem to transmit messages in a species‐independent manner, meaning that exosomes from one species are able to effectively communicate their messages, at least in part, to another species. In the present study, we revealed that exosomes derived from the blood of rats successfully transmitted the information they carried to mice and functioned effectively in mice, both therapeutically and pathogenically. This effective communication of exosome information occurs not only between rats and mice but also between humans and mice. Research has disclosed that human exosomes can effectively convey information to mice. A previous study revealed that exosomes in the serum of PD patients contain higher levels of α‐syn and inflammatory cytokines such as IL‐1β and TNF‐α than exosomes in the serum of neurologically normal controls, ultimately leading to the aggregation of α‐syn. Moreover, treatment with exosomes from PD patient serum caused dopaminergic neuron degeneration and induced microglial activation and movement defects in mice [44]. Interestingly, exosomes from healthy humans have neuroprotective effects in PD mice, such as improving motor function and reducing dopaminergic neuron degeneration [21]. These findings imply that some of the information conveyed by exosomes is universal and not limited to a particular species.

Strong evidence indicates that the benefits of exosome therapy may be related to the contents of exosomes [45, 46]. Our previous study showed that exosomes derived from healthy volunteers improve behavioral capacity, pathological symptoms, and molecular changes in a mouse model of PD. A prior study identified metabolites that are differentially expressed in exosomes from PD model mice and revealed the correlation between these metabolites and PD pathology [21]. These differentially expressed metabolites included cholesterol, nicotinic acid, neopterin, and adenine, which are enriched in the tyrosine metabolism pathway, purine metabolism pathway, and glutamate metabolism pathway. However, the mechanism by which exosomes play a therapeutic role has not been directly revealed, and the role of exosomal metabolites has not been discovered. The present study further investigated the function of exosomes in inducing physiological or pathological changes in mice through the transmission of metabolic substances. The present study showed that the peripheral and intracranial injection of adenine can significantly ameliorate the pathological features of PD and motor dysfunction in mice. Adenine, which is a kind of purine base, is the basic unit of biosynthesis and metabolism and performs numerous biochemical functions [22, 23]. Reductions in adenine levels were observed in the brain‐ and blood‐derived exosomes of PD model mice according to widely targeted metabolomics technology, and adenine supplementation had a good therapeutic effect. Both dopamine D2 receptors and adenosine A2A receptors control motor‐related neural circuits, and both appear to work by regulating cAMP accumulation [47, 48]. We further found that after adenine treatment, the levels of APRT and cAMP were increased, and PKA was activated, but A2A receptors were unaltered, indicating that adenine activates cAMP/PKA signaling via the purine synthesis remedy pathway rather than the adenosine receptor pathway. Adenine has been identified as a signaling molecule in mammals [49, 50] and serves as the primary ligand for adenine receptors (AdeR) [51, 52]. AdeR, a G protein‐coupled receptor (GPCR), currently includes rAdeR (rat), mAde1R and mAde2R (mouse), and cAdeR (hamster), and is recognized as the third family of purinergic receptors, known as P0 (P zero) receptors [53]. AdeR is highly expressed in various brain regions of rats, including the cerebral cortex and striatum, with peak expression levels observed in small neurons of the dorsal root ganglia (DRG), suggesting its role in nociception [49, 52]. It has been reported that adenine reduces the production of cAMP in mAde1R‐transfected 1321 N1 astrocytoma cells and cAdeR‐transfected CHO cells, a function that is dependent on AdeR [53]. However, the effect of adenine in triggering intracellular Ca^2+^ release was only observed in cAdeR‐transfected CHO cells, but not in mAde1R‐transfected 1321 N1 astrocytoma cells [53]. These similarities and differences may be attributed to the moderate homology among different AdeR subtypes [53]. The discovery that adenine reduces cAMP accumulation diverges from our findings in the brain, and we hypothesize that this may be associated with the complex pathological state of PD. The effects of adenine in the brain of mice are the collective outcomes of responses from multiple brain regions and cell types, potentially distinguishing them from the outcomes observed in a single cell line. Furthermore, the effects of adenine in vivo encompass a variety of metabolic processes, not limited solely to AdeR, but also including purine synthesis salvage pathways, among others. Meanwhile, the effects of adenine in vivo encompass a variety of metabolic processes, not only limited to AdeR activation, but also involve purine synthesis remedy pathways. Consequently, the specific metabolic pathways of adenine in the pathology of PD remain unclear and necessitate further investigation. The regulation of cAMP/PKA signaling by adenine is also related to iron metabolism. Studies have shown that adenine, as an inducer of ferrmodulin, can reduce iron overload in mice through cAMP/PKA‐mediated modulation of the BMP/SMAD signaling pathway [24]. Although iron metabolism and ferroptosis have been reported to contribute to the pathogenesis of diseases, such as ischemia/reperfusion, and neurodegenerative diseases, such as Alzheimer's disease and PD, there is currently no evidence to indicate whether adenine is therefore involved in PD [54, 55].

With the identification of the adenine and cAMP/PKA signaling pathways, the underlying mechanism by which exosomes are involved in PD was revealed. Although this study provides evidence for the etiology and treatment of PD, it is still difficult to use single exosomal metabolites as therapeutic agents in clinical treatment. In addition to metabolites, other informational molecules in exosomes, such as proteins and nucleic acids, are also important signal transmitters as research objects.

Taken together, the results of this study demonstrate the therapeutic effects of exosomes derived from the blood of healthy rats and the pathogenic effects of exosomes derived from the blood of PD model rats on motor coordination and PD pathology. Moreover, a new mechanism underlying exosome‐mediated communication in PD has been demonstrated: adenine alleviates motor coordination disorder and dopaminergic neuron loss and maintains the homeostasis of oxidative stress and neuroinflammation by activating cAMP/PKA signaling in PD. Our research provides novel insights into the role of exosomes in the etiology and treatment of PD and illustrates the mechanism by which exosomes are involved in PD pathophysiology by transmitting adenine.

## Author Contributions

**Lei Chen:** methodology, writing – revised draft preparation, validation, data curation, visualization. **Yi‐Ting Shao:** methodology, data curation. **Ji Geng:** validation, visualization. **Hua Liu:** validation, data curation, visualization. **Qing‐Shan Liu:** supervision. **Yong Cheng:** conceptualization, funding acquisition, writing – reviewing and editing. **Ting Sun:** methodology, writing – original draft preparation, funding acquisition. All authors read and approved the final manuscript.

## Ethical Approval and Consent to Participate

The Animal Care and Use Committee of the Minzu University of China approved all the experiments in this study.

## Conflicts of Interest

The authors declare no conflicts of interest.

## Supporting information


**Figure S1.** Full unedited gel/blot for Figures.

## Data Availability

The data that support the findings of this study are available from the corresponding author upon reasonable request.

## References

[1] A. Ascherio and M. A. Schwarzschild , “The Epidemiology of Parkinson's Disease: Risk Factors and Prevention,” Lancet Neurology 15, no. 12 (2016): 1257–1272. 27751556 10.1016/S1474-4422(16)30230-7

[2] D. Aarsland , L. Batzu , G. M. Halliday , et al., “Parkinson Disease‐Associated Cognitive Impairment,” Nature Reviews. Disease Primers 7, no. 1 (2021): 47. 10.1038/s41572-021-00280-3 34210995

[3] E. Tolosa , A. Garrido , S. W. Scholz , and W. Poewe , “Challenges in the Diagnosis of Parkinson's Disease,” Lancet Neurology 20, no. 5 (2021): 385–397. 33894193 10.1016/S1474-4422(21)00030-2 PMC8185633

[4] N. Vijiaratnam , T. Simuni , O. Bandmann , H. R. Morris , and T. Foltynie , “Progress Towards Therapies for Disease Modification in Parkinson's Disease,” Lancet Neurology 20, no. 7 (2021): 559–572. 34146514 10.1016/S1474-4422(21)00061-2

[5] L. V. Kalia and A. E. Lang , “Parkinson's Disease,” Lancet 386, no. 9996 (2015): 896–912. 25904081 10.1016/S0140-6736(14)61393-3

[6] S. Liu , S. Wang , R. Gu , et al., “The XPO1 Inhibitor KPT‐8602 Ameliorates Parkinson's Disease by Inhibiting the NF‐κB/NLRP3 Pathway,” Frontiers in Pharmacology 13 (2022): 847605. 35721113 10.3389/fphar.2022.847605 PMC9200340

[7] T. Sun , L. Chen , R. Liu , Q. S. Liu , and Y. Cheng , “Sophora Alopecuroides Alleviates Neuroinflammation and Oxidative Damage of Parkinson's Disease In Vitro and In Vivo,” American Journal of Chinese Medicine 51, no. 2 (2023): 309–328. 36611142 10.1142/S0192415X23500167

[8] C. He , S. Zheng , Y. Luo , and B. Wang , “Exosome Theranostics: Biology and Translational Medicine,” Theranostics 8, no. 1 (2018): 237–255. 29290805 10.7150/thno.21945 PMC5743472

[9] R. Kalluri and V. S. LeBleu , “The Biology, Function, and Biomedical Applications of Exosomes,” Science 367, no. 6478 (2020): eaau6977, 10.1126/science.aau6977. 32029601 PMC7717626

[10] M. Cully , “Exosome‐Based Candidates Move Into the Clinic,” Nature Reviews. Drug Discovery 20, no. 1 (2021): 6–7. 10.1038/d41573-020-00220-y 33311580

[11] J. Meldolesi , “Exosomes and Ectosomes in Intercellular Communication,” Current Biology 28, no. 8 (2018): R435–R444. 29689228 10.1016/j.cub.2018.01.059

[12] E. van der Pol , A. N. Böing , P. Harrison , A. Sturk , and R. Nieuwland , “Classification, Functions, and Clinical Relevance of Extracellular Vesicles,” Pharmacological Reviews 64, no. 3 (2012): 676–705. 22722893 10.1124/pr.112.005983

[13] R. N. Hamzah , K. M. Alghazali , A. S. Biris , and R. J. Griffin , “Exosome Traceability and Cell Source Dependence on Composition and Cell‐Cell Cross Talk,” International Journal of Molecular Sciences 22, no. 10 (2021): 5346, 10.3390/ijms22105346. 34069542 PMC8161017

[14] Y. Liu , Y. Li , J. Zang , et al., “CircOGDH Is a Penumbra Biomarker and Therapeutic Target in Acute Ischemic Stroke,” Circulation Research 130, no. 6 (2022): 907–924. 35189704 10.1161/CIRCRESAHA.121.319412

[15] T. Zhang , S. Ma , J. Lv , et al., “The Emerging Role of Exosomes in Alzheimer's Disease,” Ageing Research Reviews 68 (2021): 101321. 33727157 10.1016/j.arr.2021.101321

[16] Y. Du , R. Qiu , L. Chen , et al., “Identification of Serum Exosomal Metabolomic and Proteomic Profiles for Remote Ischemic Preconditioning,” Journal of Translational Medicine 21, no. 1 (2023): 241. 37009888 10.1186/s12967-023-04070-1 PMC10069038

[17] J. Chen and M. Chopp , “Exosome Therapy for Stroke,” Stroke 49, no. 5 (2018): 1083–1090. 29669873 10.1161/STROKEAHA.117.018292 PMC6028936

[18] H. Asai , S. Ikezu , S. Tsunoda , et al., “Depletion of Microglia and Inhibition of Exosome Synthesis Halt Tau Propagation,” Nature Neuroscience 18, no. 11 (2015): 1584–1593, 10.1038/nn.4132. 26436904 PMC4694577

[19] Y. Du , W. L. Tan , L. Chen , et al., “Exosome Transplantation From Patients With Schizophrenia Causes Schizophrenia‐Relevant Behaviors in Mice: An Integrative Multi‐Omics Data Analysis,” Schizophrenia Bulletin 47, no. 5 (2021): 1288–1299. 33837780 10.1093/schbul/sbab039 PMC8379541

[20] Z. X. Wei , G. J. Xie , X. Mao , et al., “Exosomes From Patients With Major Depression Cause Depressive‐Like Behaviors in Mice With Involvement of miR‐139‐5p‐Regulated Neurogenesis,” Neuropsychopharmacology 45, no. 6 (2020): 1050–1058. 31986519 10.1038/s41386-020-0622-2 PMC7162931

[21] T. Sun , Z. X. Ding , X. Luo , Q. S. Liu , and Y. Cheng , “Blood Exosomes Have Neuroprotective Effects in a Mouse Model of Parkinson's Disease,” Oxidative Medicine and Cellular Longevity 2020 (2020): 3807476. 33294121 10.1155/2020/3807476 PMC7714585

[22] H. Heyn and M. Esteller , “An Adenine Code for DNA: A Second Life for N6‐Methyladenine,” Cell 161, no. 4 (2015): 710–713. 25936836 10.1016/j.cell.2015.04.021

[23] A. M. Cardoso , M. R. Schetinger , P. Correia‐de‐Sá , and J. Sévigny , “Impact of Ectonucleotidases in Autonomic Nervous Functions,” Autonomic Neuroscience 191 (2015): 25–38. 26008223 10.1016/j.autneu.2015.04.014

[24] Y. Zhang , X. Wang , Q. Wu , et al., “Adenine Alleviates Iron Overload by cAMP/PKA Mediated Hepatic Hepcidin in Mice,” Journal of Cellular Physiology 233, no. 9 (2018): 7268–7278. 29600572 10.1002/jcp.26559 PMC7754534

[25] Y. Salomon , “Cellular Responsiveness to Hormones and Neurotransmitters: Conversion of [3H]Adenine to [3H]cAMP in Cell Monolayers, Cell Suspensions, and Tissue Slices,” Methods in Enzymology 195 (1991): 22–28. 1851928 10.1016/0076-6879(91)95151-9

[26] R. M. Essam and E. A. Kandil , “P‐CREB and p‐DARPP‐32 Orchestrating the Modulatory Role of cAMP/PKA Signaling Pathway Enhanced by Roflumilast in Rotenone‐Induced Parkinson's Disease in Rats,” Chemico‐Biological Interactions 372 (2023): 110366. 36706892 10.1016/j.cbi.2023.110366

[27] D. Ballardin , L. Makrini‐Maleville , A. Seper , E. Valjent , and H. Rebholz , “5‐HT4R Agonism Reduces L‐DOPA‐Induced Dyskinesia Via Striatopallidal Neurons in Unilaterally 6‐OHDA Lesioned Mice,” Neurobiology of Disease 198 (2024): 106559. 38852753 10.1016/j.nbd.2024.106559

[28] Y. Du , Y. Yu , Y. Hu , et al., “Genome‐Wide, Integrative Analysis Implicates Exosome‐Derived MicroRNA Dysregulation in Schizophrenia,” Schizophrenia Bulletin 45, no. 6 (2019): 1257–1266. 30770930 10.1093/schbul/sby191 PMC6811837

[29] L. J. Vella , B. J. Scicluna , L. Cheng , et al., “A Rigorous Method to Enrich for Exosomes From Brain Tissue,” Journal of Extracellular Vesicles 6, no. 1 (2017): 1348885. 28804598 10.1080/20013078.2017.1348885 PMC5533148

[30] C. Théry , K. W. Witwer , E. Aikawa , et al., “Minimal Information for Studies of Extracellular Vesicles 2018 (MISEV2018): A Position Statement of the International Society for Extracellular Vesicles and Update of the MISEV2014 Guidelines,” Journal of Extracellular Vesicles 7, no. 1 (2018): 1535750. 30637094 10.1080/20013078.2018.1535750 PMC6322352

[31] Y. Liang , L. Duan , J. Lu , and J. Xia , “Engineering Exosomes for Targeted Drug Delivery,” Theranostics 11, no. 7 (2021): 3183–3195. 33537081 10.7150/thno.52570 PMC7847680

[32] L. Milane , A. Singh , G. Mattheolabakis , M. Suresh , and M. M. Amiji , “Exosome Mediated Communication Within the Tumor Microenvironment,” Journal of Controlled Release 219 (2015): 278–294. 26143224 10.1016/j.jconrel.2015.06.029

[33] L. Zhang and D. Yu , “Exosomes in Cancer Development, Metastasis, and Immunity,” Biochimica Et Biophysica Acta. Reviews on Cancer 1871, no. 2 (2019): 455–468. 31047959 10.1016/j.bbcan.2019.04.004 PMC6542596

[34] M. Ghasemi , E. Roshandel , M. Mohammadian , B. Farhadihosseinabadi , P. Akbarzadehlaleh , and K. Shamsasenjan , “Mesenchymal Stromal Cell‐Derived Secretome‐Based Therapy for Neurodegenerative Diseases: Overview of Clinical Trials,” Stem Cell Research & Therapy 14, no. 1 (2023): 122. 37143147 10.1186/s13287-023-03264-0 PMC10161443

[35] R. Upadhya , W. Zingg , S. Shetty , and A. K. Shetty , “Astrocyte‐Derived Extracellular Vesicles: Neuroreparative Properties and Role in the Pathogenesis of Neurodegenerative Disorders,” Journal of Controlled Release 323 (2020): 225–239. 32289328 10.1016/j.jconrel.2020.04.017 PMC7299747

[36] M. Guo , J. Wang , Y. Zhao , et al., “Microglial Exosomes Facilitate α‐Synuclein Transmission in Parkinson's Disease,” Brain 143, no. 5 (2020): 1476–1497. 32355963 10.1093/brain/awaa090 PMC7241957

[37] R. Kojima , D. Bojar , G. Rizzi , et al., “Designer Exosomes Produced by Implanted Cells Intracerebrally Deliver Therapeutic Cargo for Parkinson's Disease Treatment,” Nature Communications 9, no. 1 (2018): 1305, 10.1038/s41467-018-03733-8. PMC5880805 29610454

[38] S. Dutta , S. Hornung , H. B. Taha , and G. Bitan , “Biomarkers for Parkinsonian Disorders in CNS‐Originating EVs: Promise and Challenges,” Acta Neuropathologica 145, no. 5 (2023): 515–540. 37012443 10.1007/s00401-023-02557-1 PMC10071251

[39] Y. Deng , R. Duan , W. Ding , et al., “Astrocyte‐Derived Exosomal Nicotinamide Phosphoribosyltransferase (Nampt) Ameliorates Ischemic Stroke Injury by Targeting AMPK/mTOR Signaling to Induce Autophagy,” Cell Death & Disease 13, no. 12 (2022): 1057. 36539418 10.1038/s41419-022-05454-9 PMC9767935

[40] L. Leggio , F. L'Episcopo , A. Magrì , et al., “Small Extracellular Vesicles Secreted by Nigrostriatal Astrocytes Rescue Cell Death and Preserve Mitochondrial Function in Parkinson's Disease,” Advanced Healthcare Materials 11, no. 20 (2022): e2201203. 35856921 10.1002/adhm.202201203 PMC11468249

[41] C. Peng , J. Q. Trojanowski , and V. M. Lee , “Protein transmission in neurodegenerative disease,” Nature Reviews. Neurology 16, no. 4 (2020): 199–212. 32203399 10.1038/s41582-020-0333-7 PMC9242841

[42] H. Wei , Q. Chen , L. Lin , et al., “Regulation of Exosome Production and Cargo Sorting,” International Journal of Biological Sciences 17, no. 1 (2021): 163–177. 33390841 10.7150/ijbs.53671 PMC7757038

[43] Y. Huang , Z. Liu , N. Li , et al., “Parkinson's Disease Derived Exosomes Aggravate Neuropathology in SNCA*A53T Mice,” Annals of Neurology 92, no. 2 (2022): 230–245. 35596947 10.1002/ana.26421

[44] C. Han , N. Xiong , X. Guo , et al., “Exosomes From Patients with Parkinson's Disease Are Pathological in Mice,” Journal of Molecular Medicine (Berlin, Germany) 97, no. 9 (2019): 1329–1344. 31302715 10.1007/s00109-019-01810-z

[45] A. de Rus Jacquet , J. L. Tancredi , A. L. Lemire , M. C. DeSantis , W. P. Li , and E. K. O'Shea , “The LRRK2 G2019S Mutation Alters Astrocyte‐To‐Neuron Communication via Extracellular Vesicles and Induces Neuron Atrophy in a Human iPSC‐Derived Model of Parkinson's Disease,” eLife 10 (2021): e73062. 34590578 10.7554/eLife.73062 PMC8514240

[46] H. X. Chen , F. C. Liang , P. Gu , et al., “Exosomes Derived From Mesenchymal Stem Cells Repair a Parkinson's Disease Model by Inducing Autophagy,” Cell Death & Disease 11, no. 4 (2020): 288. 32341347 10.1038/s41419-020-2473-5 PMC7184757

[47] T. N. Lerner and A. C. Kreitzer , “RGS4 Is Required for Dopaminergic Control of Striatal LTD and Susceptibility to Parkinsonian Motor Deficits,” Neuron 73, no. 2 (2012): 347–359. 22284188 10.1016/j.neuron.2011.11.015 PMC3269032

[48] M. E. Ferrero , “Purinoceptors in Inflammation: Potential as Anti‐Inflammatory Therapeutic Targets,” Frontiers in Bioscience (Landmark Ed) 16, no. 6 (2011): 2172–2186. 10.2741/3846 21622169

[49] E. Bender , A. Buist , M. Jurzak , et al., “Characterization of an Orphan G Protein‐Coupled Receptor Localized in the Dorsal Root Ganglia Reveals Adenine as a Signaling Molecule,” Proceedings of the National Academy of Sciences of the United States of America 99, no. 13 (2002): 8573–8578. 12084918 10.1073/pnas.122016499 PMC124315

[50] T. Borrmann , A. Abdelrahman , R. Volpini , et al., “Structure‐Activity Relationships of Adenine and Deazaadenine Derivatives as Ligands for Adenine Receptors, a New Purinergic Receptor Family,” Journal of Medicinal Chemistry 52, no. 19 (2009): 5974–5989. 19731917 10.1021/jm9006356

[51] I. von Kügelgen , A. C. Schiedel , K. Hoffmann , B. B. Alsdorf , A. Abdelrahman , and C. E. Müller , “Cloning and Functional Expression of a Novel Gi Protein‐Coupled Receptor for Adenine From Mouse Brain,” Molecular Pharmacology 73, no. 2 (2008): 469–477. 17975009 10.1124/mol.107.037069

[52] S. Gorzalka , S. Vittori , R. Volpini , G. Cristalli , I. von Kügelgen , and C. E. Müller , “Evidence for the Functional Expression and Pharmacological Characterization of Adenine Receptors in Native Cells and Tissues,” Molecular Pharmacology 67, no. 3 (2005): 955–964. 15604413 10.1124/mol.104.006601

[53] D. Thimm , M. Knospe , A. Abdelrahman , et al., “Characterization of New G Protein‐Coupled Adenine Receptors in Mouse and Hamster,” Purinergic Signal 9, no. 3 (2013): 415–426. 23608776 10.1007/s11302-013-9360-9 PMC3757137

[54] H. F. Yan , T. Zou , Q. Z. Tuo , et al., “Ferroptosis: Mechanisms and Links With Diseases,” Signal Transduction and Targeted Therapy 6, no. 1 (2021): 49. 33536413 10.1038/s41392-020-00428-9 PMC7858612

[55] F. A. Zucca , J. Segura‐Aguilar , E. Ferrari , et al., “Interactions of Iron, Dopamine and Neuromelanin Pathways in Brain Aging and Parkinson's Disease,” Progress in Neurobiology 155 (2017): 96–119. 26455458 10.1016/j.pneurobio.2015.09.012 PMC4826627

