# Supplementary material for: Dual Role of Exosomes in Parkinson's Disease: Adenine Exerts a Beneficial Effect
Source: CNS Neurosci Ther. 2025 Apr 16;31(4):e70331. doi: 10.1111/cns.70331 (PMC12001426; doi:10.1111/cns.70331)

**Supplemental Files**

**Full Unedited Gel/Blot for Figure 1C**


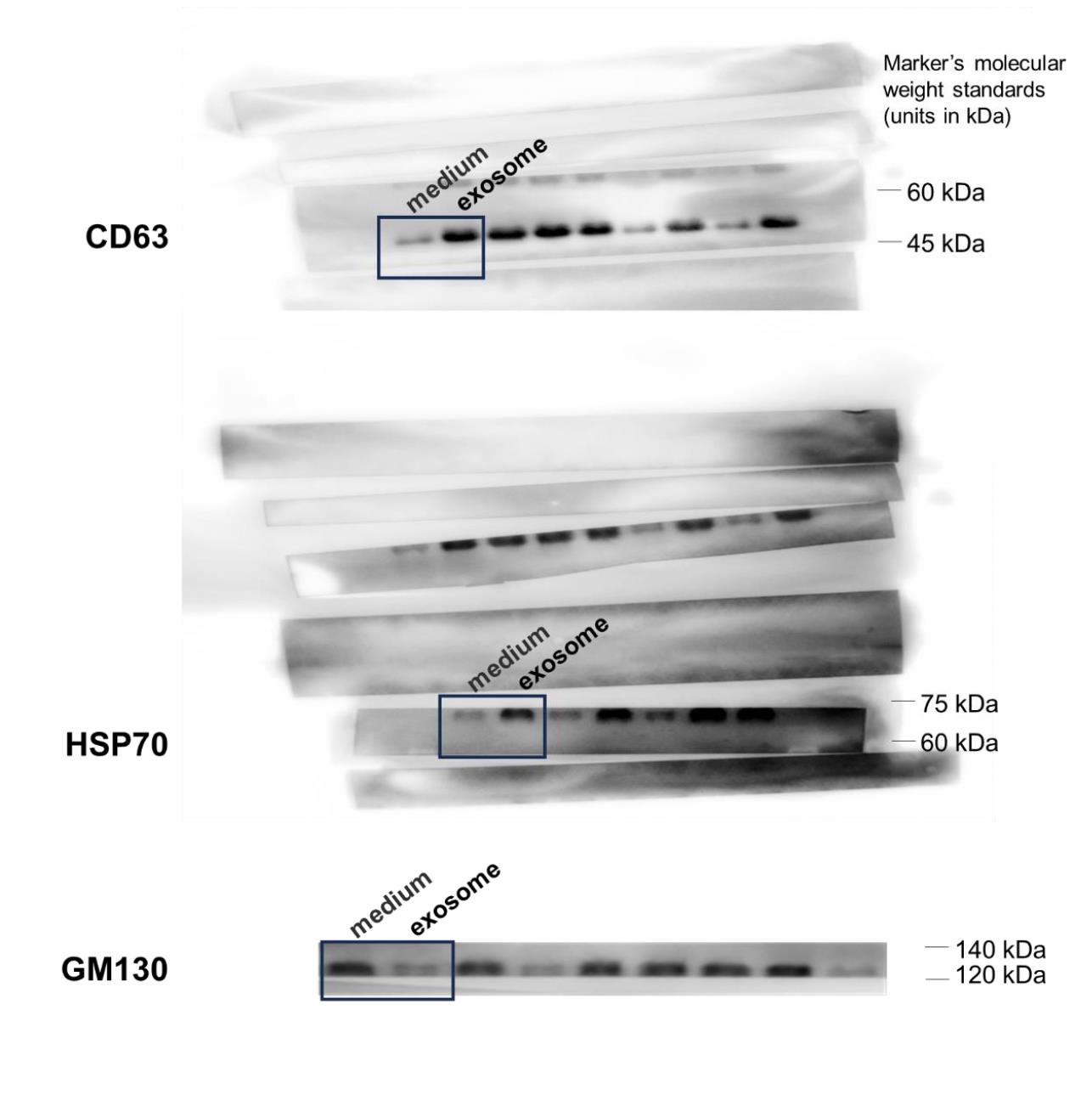

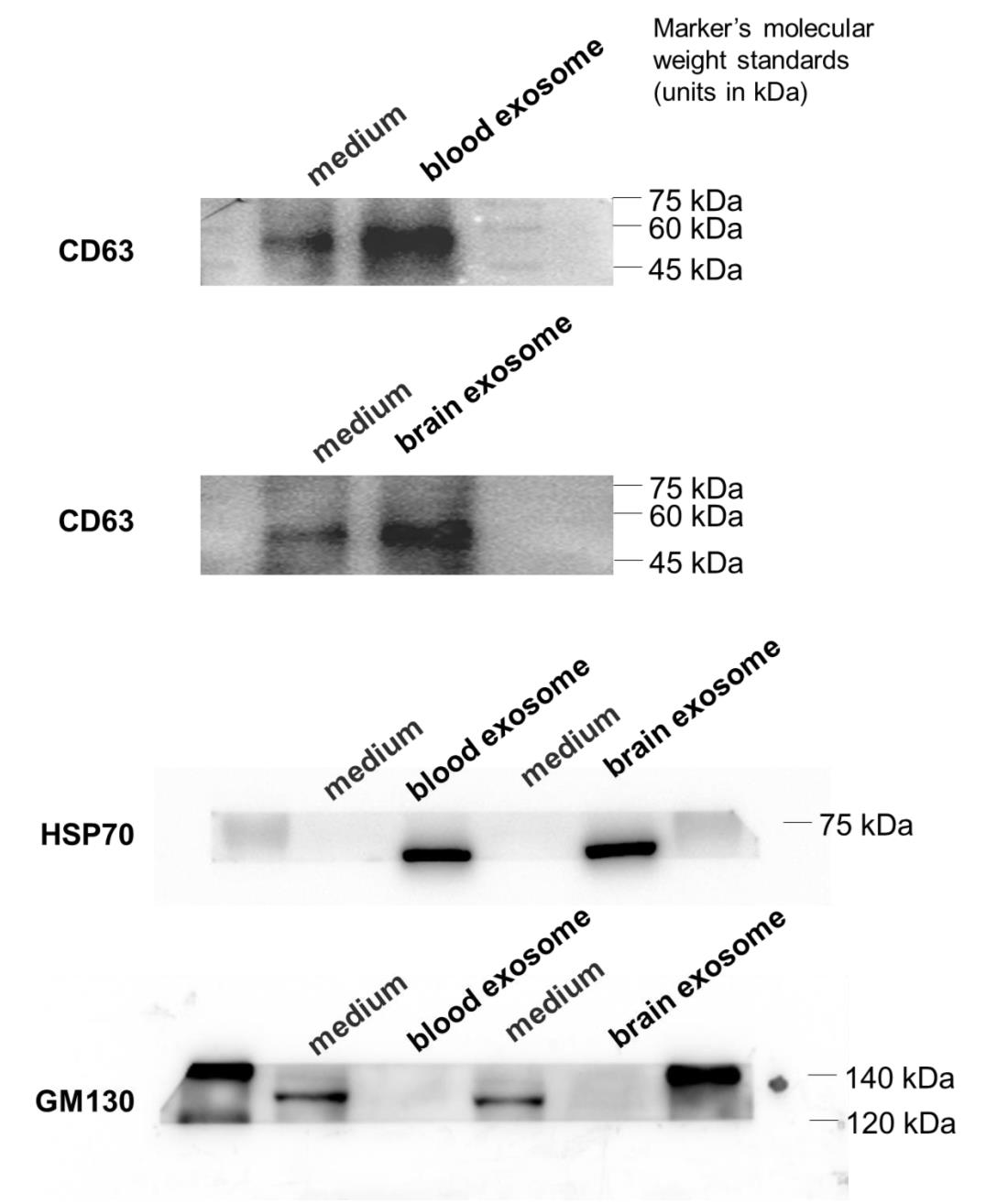


**Full Unedited Gel/Blot for Figure 2G**


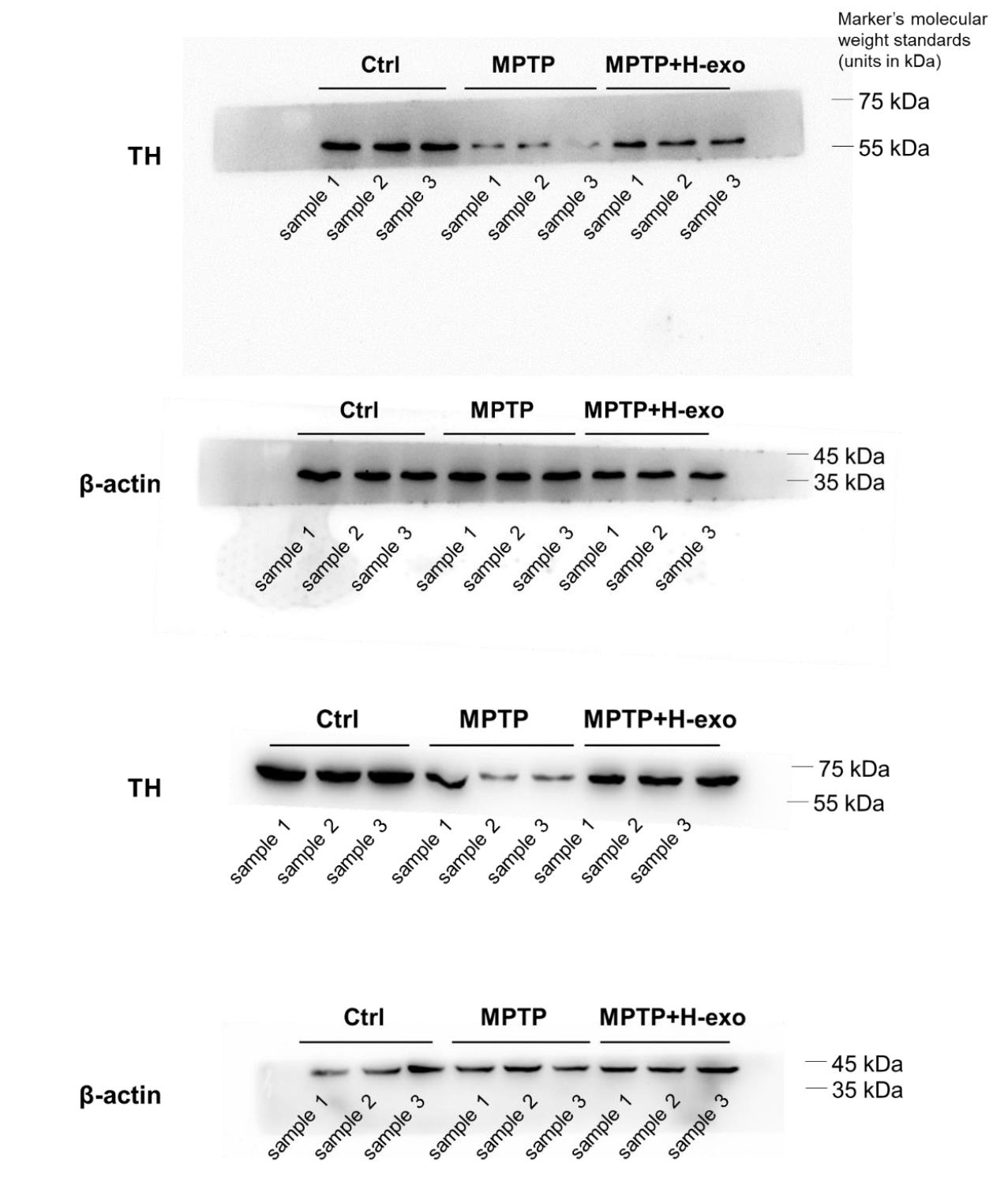


**Full Unedited Gel/Blot for Figure 3G**


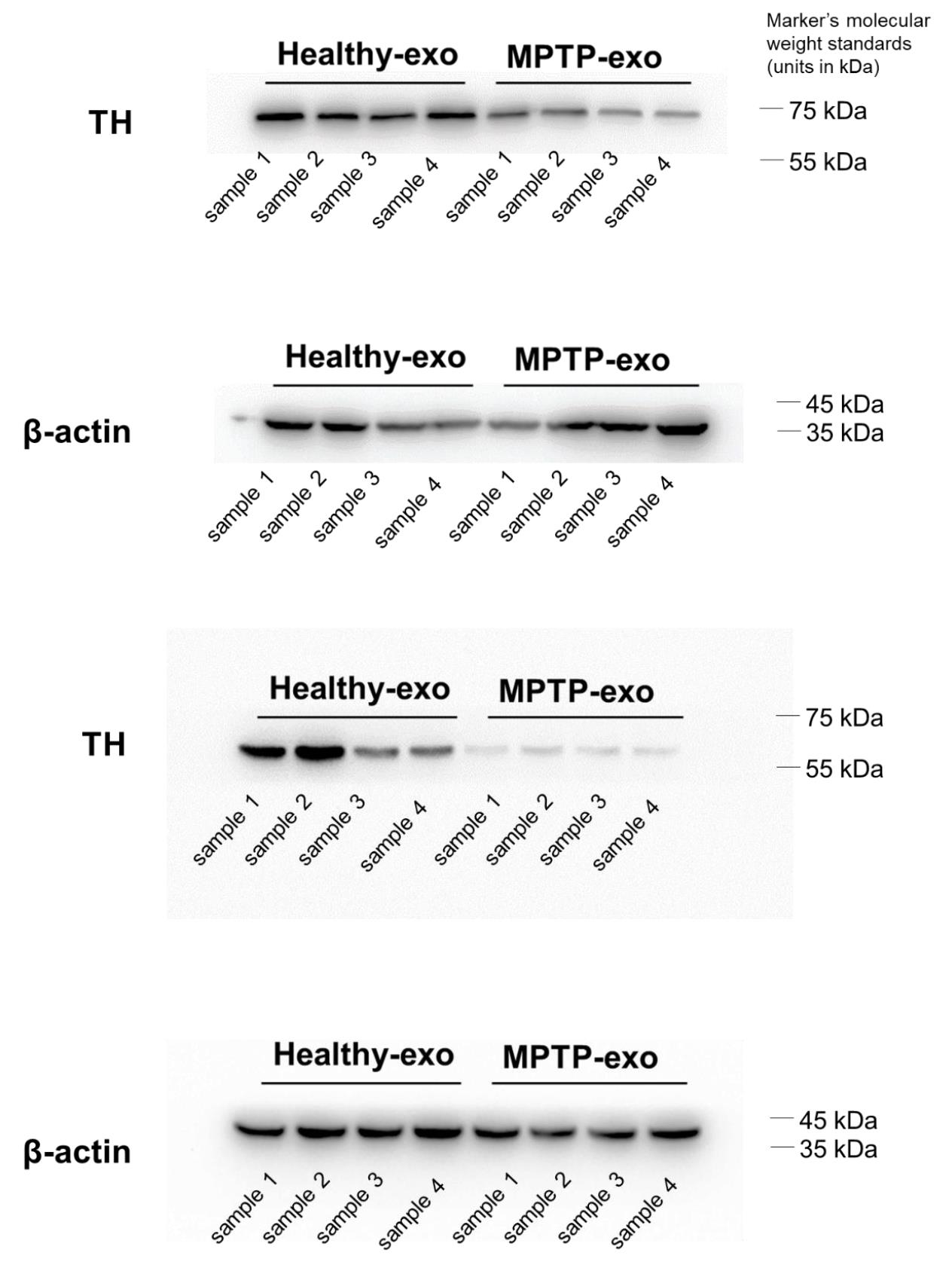


**Full Unedited Gel/Blot for Figure 4J**


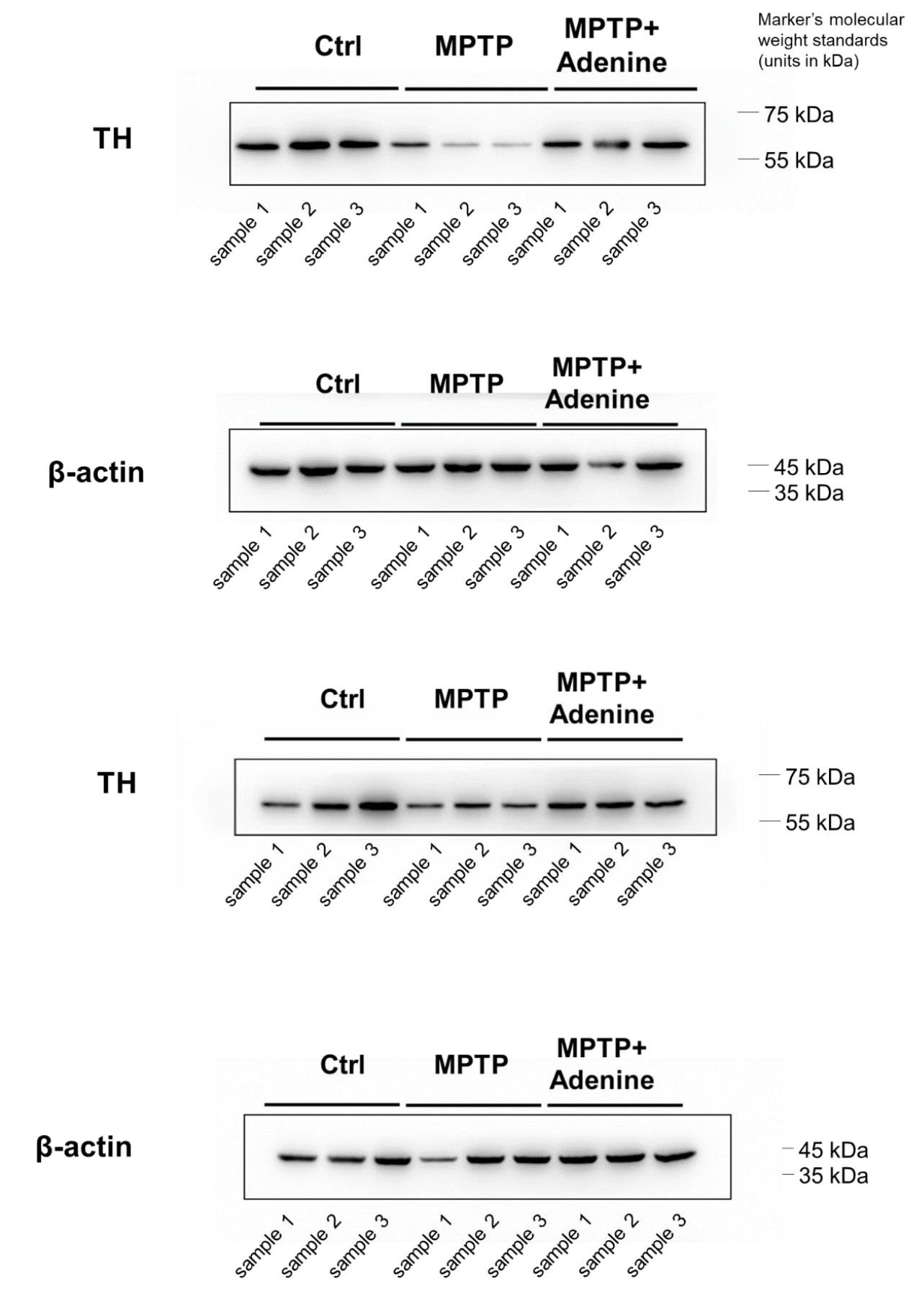


**Full Unedited Gel/Blot for Figure 5G**


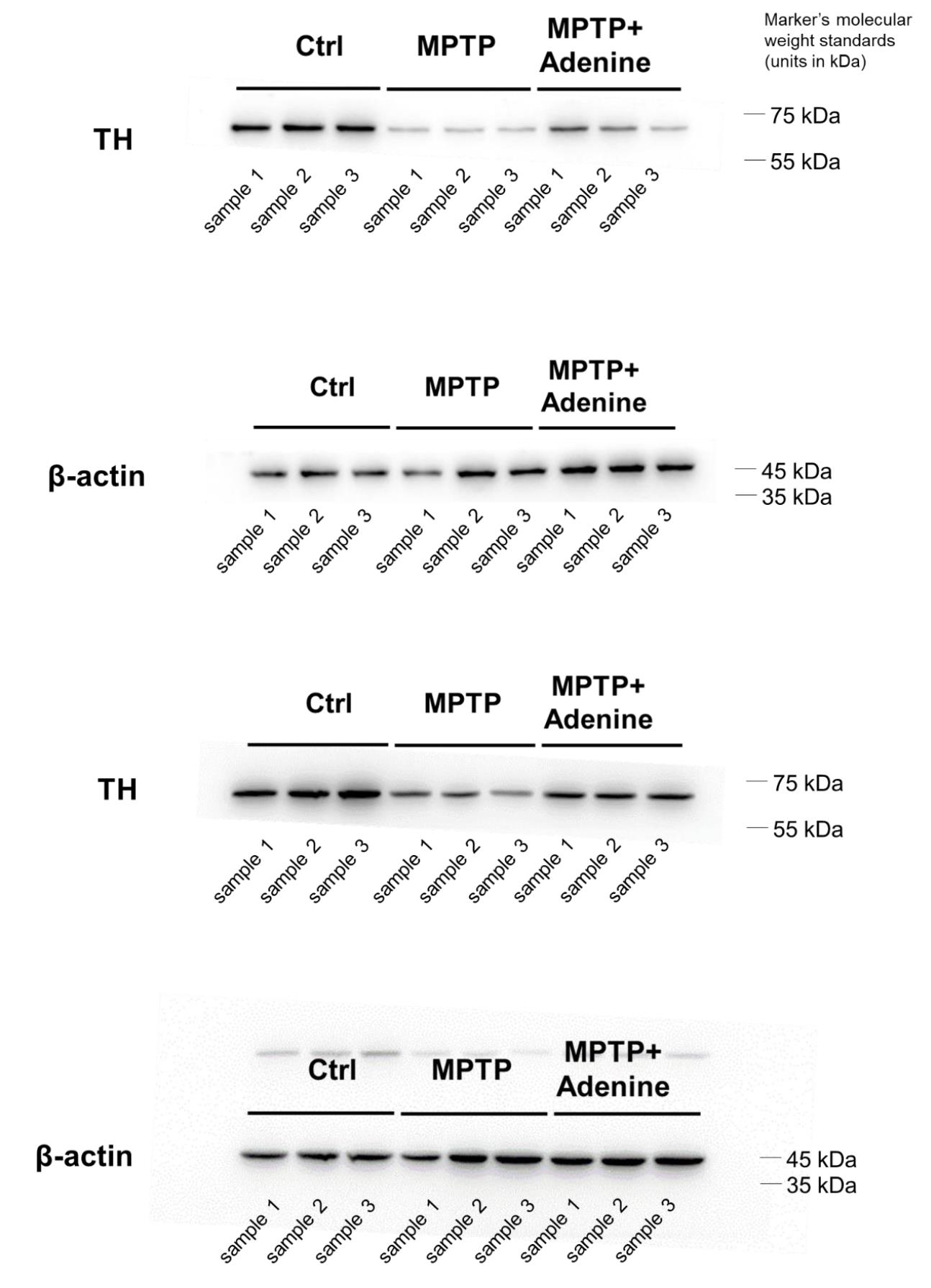


**Full Unedited Gel/Blot for Figure 6A**


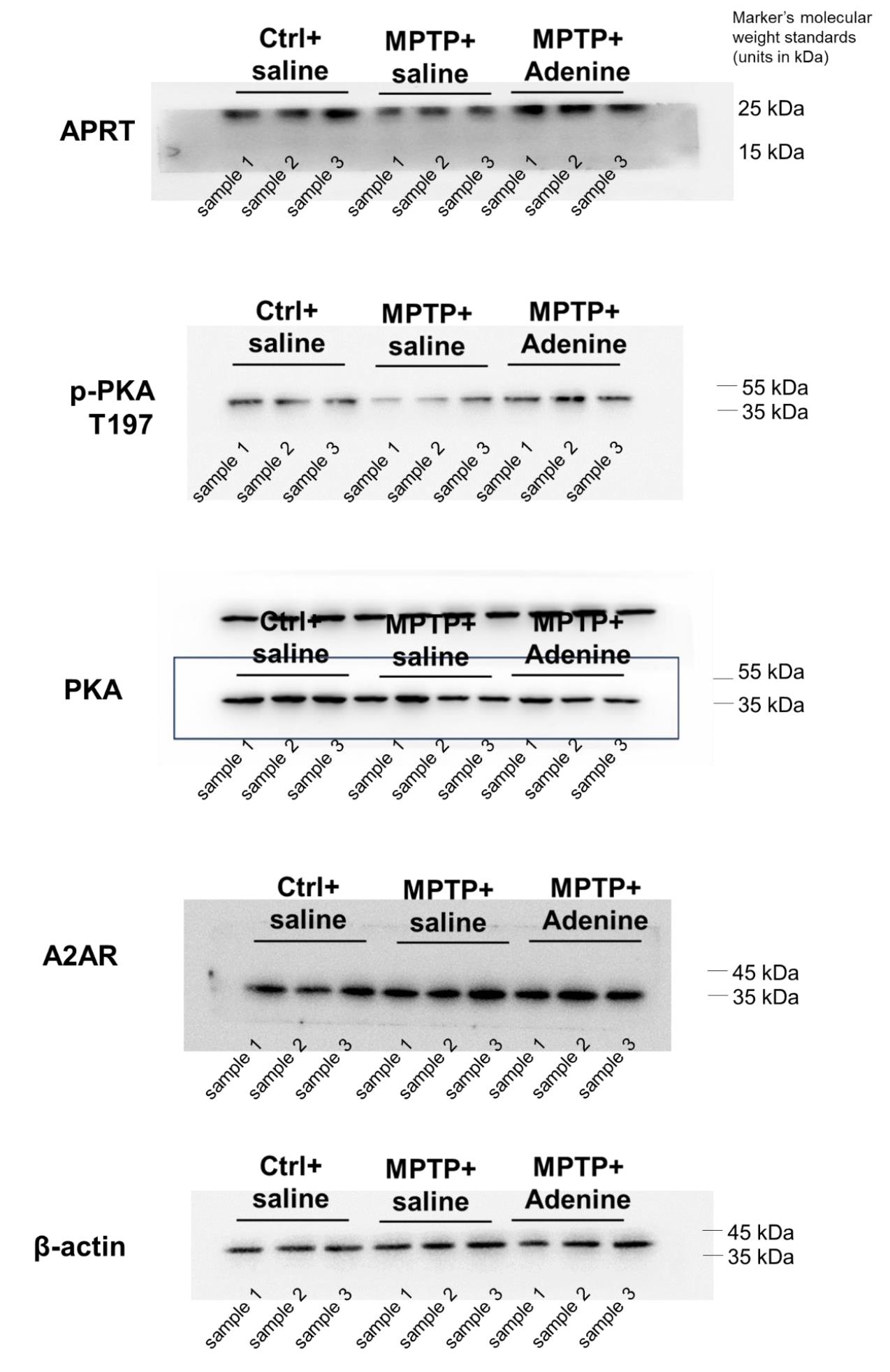


**Full Unedited Gel/Blot for Figure 6F**


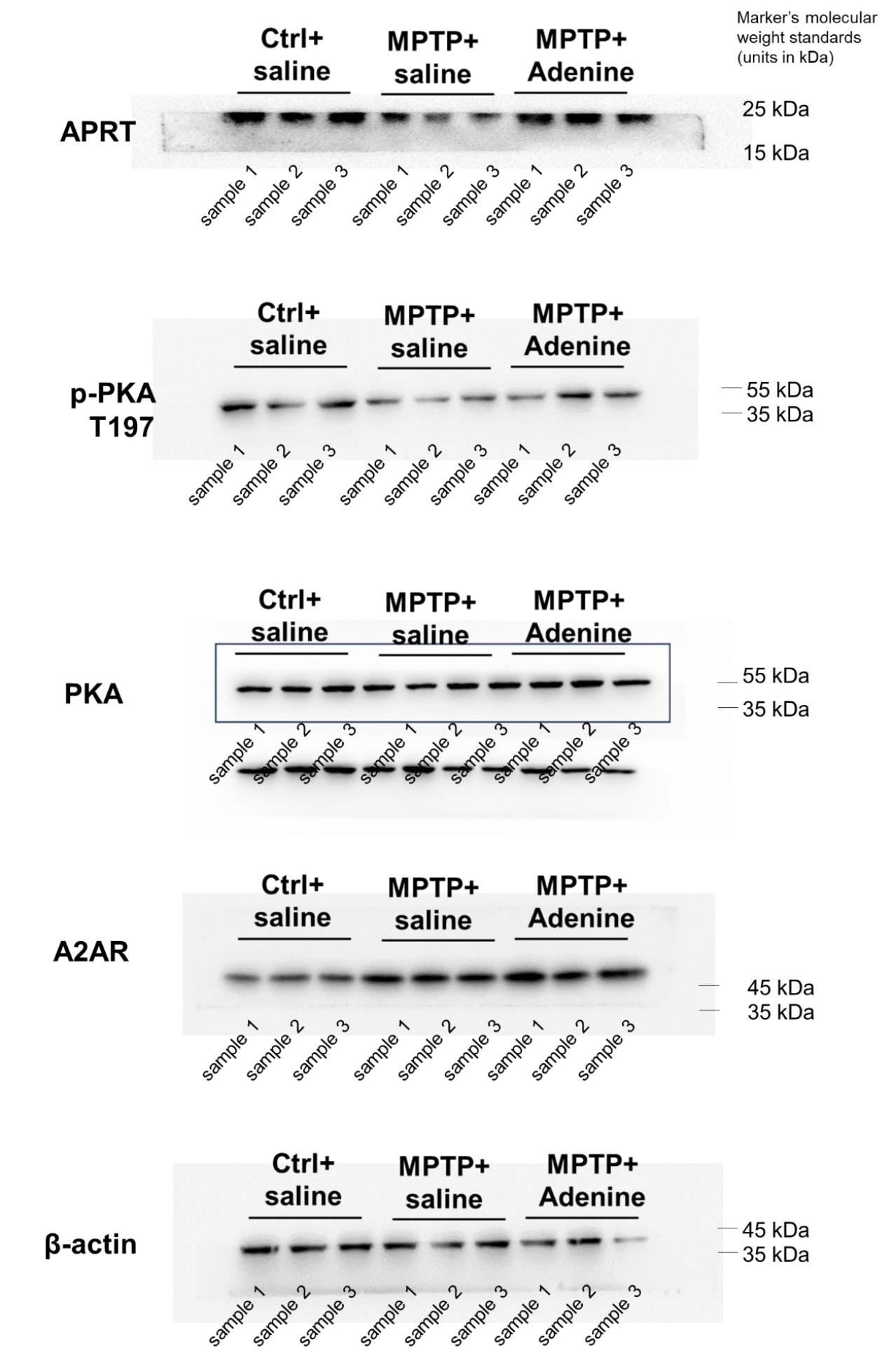

Supplement: Supplementary file 1 — Figure S1. Full unedited gel/blot for Figures. [file CNS-31-e70331-s001.zip › cns70331-sup-0001-Revised Supplemental Files -revision 2.docx]
