# Supplementary material for: Dual Role of Exosomes in Parkinson's Disease: Adenine Exerts a Beneficial Effect
Source: CNS Neurosci Ther. 2025 Apr 16;31(4):e70331. doi: 10.1111/cns.70331 (PMC12001426; doi:10.1111/cns.70331)

Supplemental Files

Full Unedited Gel/Blot for Figure 1C

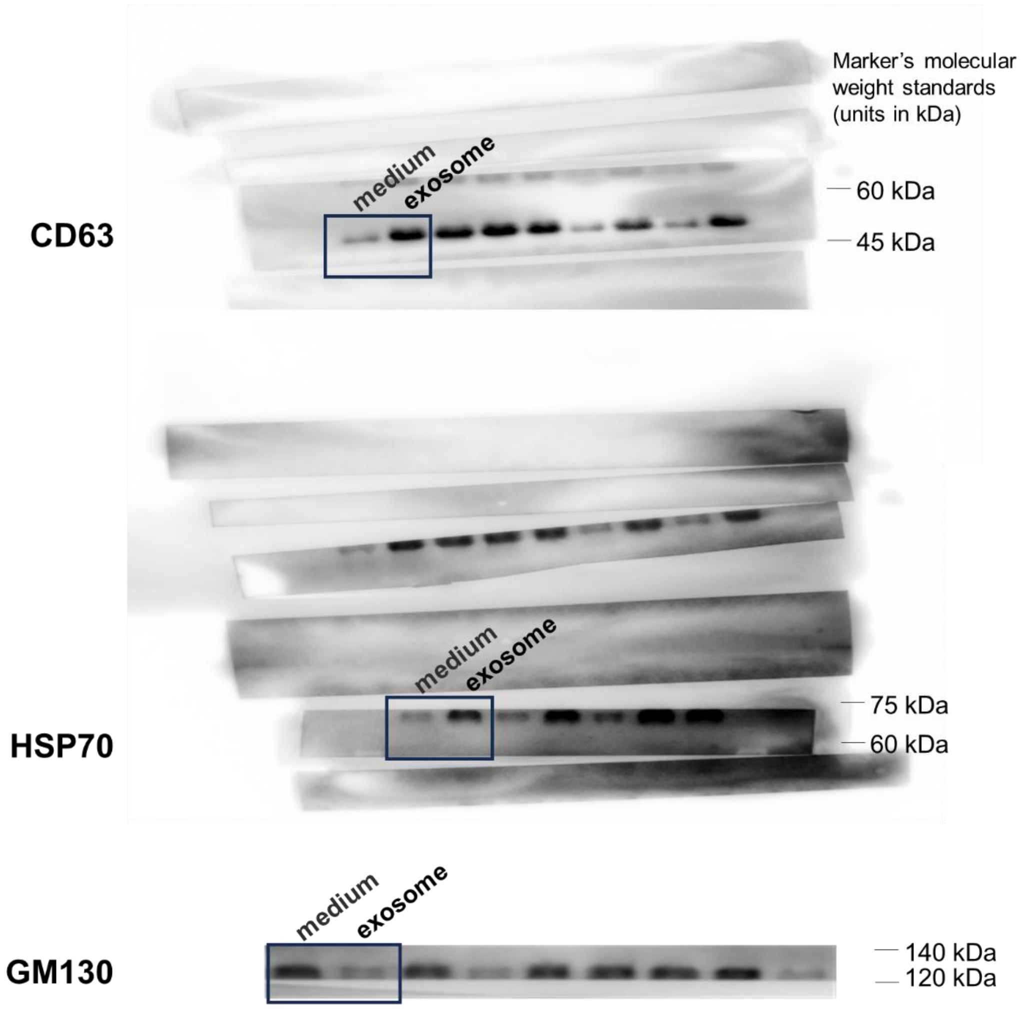

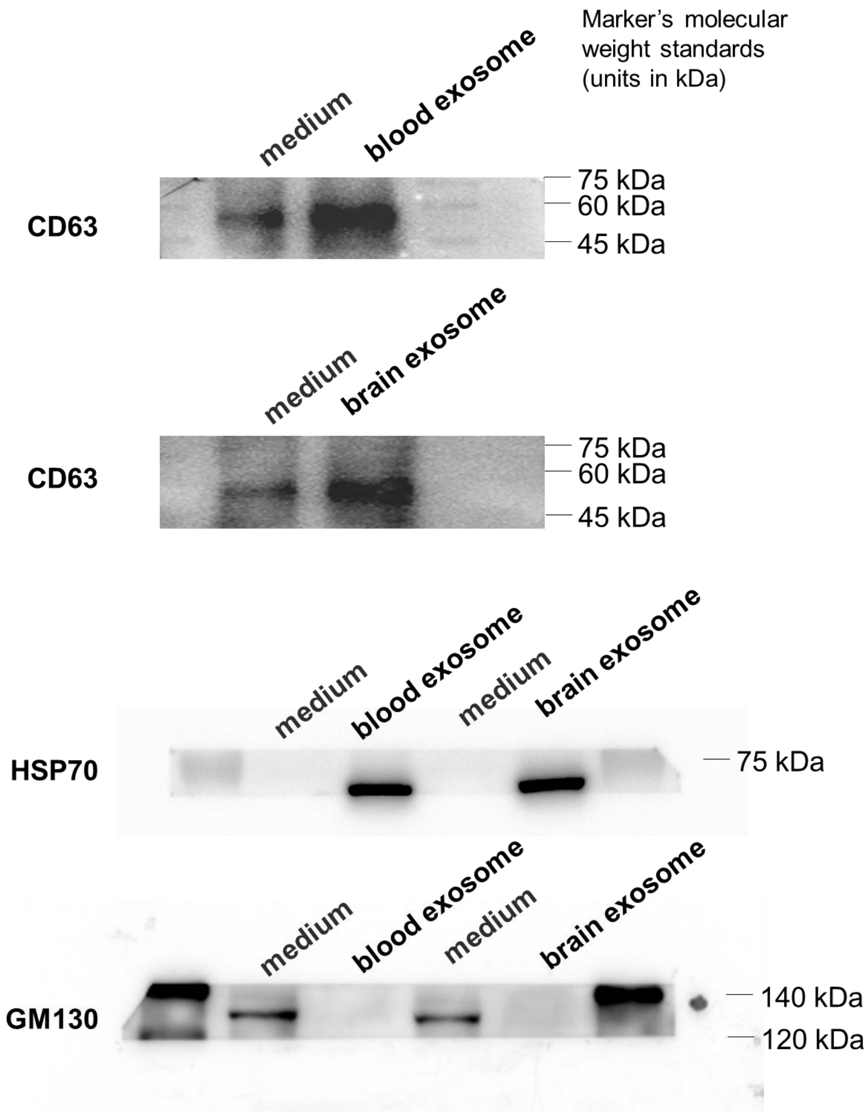
Marker's molecular weight standards (units in kDa)
medium
blood exosome
CD63
75 kDa
60 kDa
45 kDa
medium
brain exosome
CD63
75 kDa
60 kDa
45 kDa
medium
blood exosome
medium
brain exosome
HSP70
75 kDa
medium
blood exosome
medium
brain exosome
GM130
140 kDa
120 kDa

Full Unedited Gel/Blot for Figure 2G

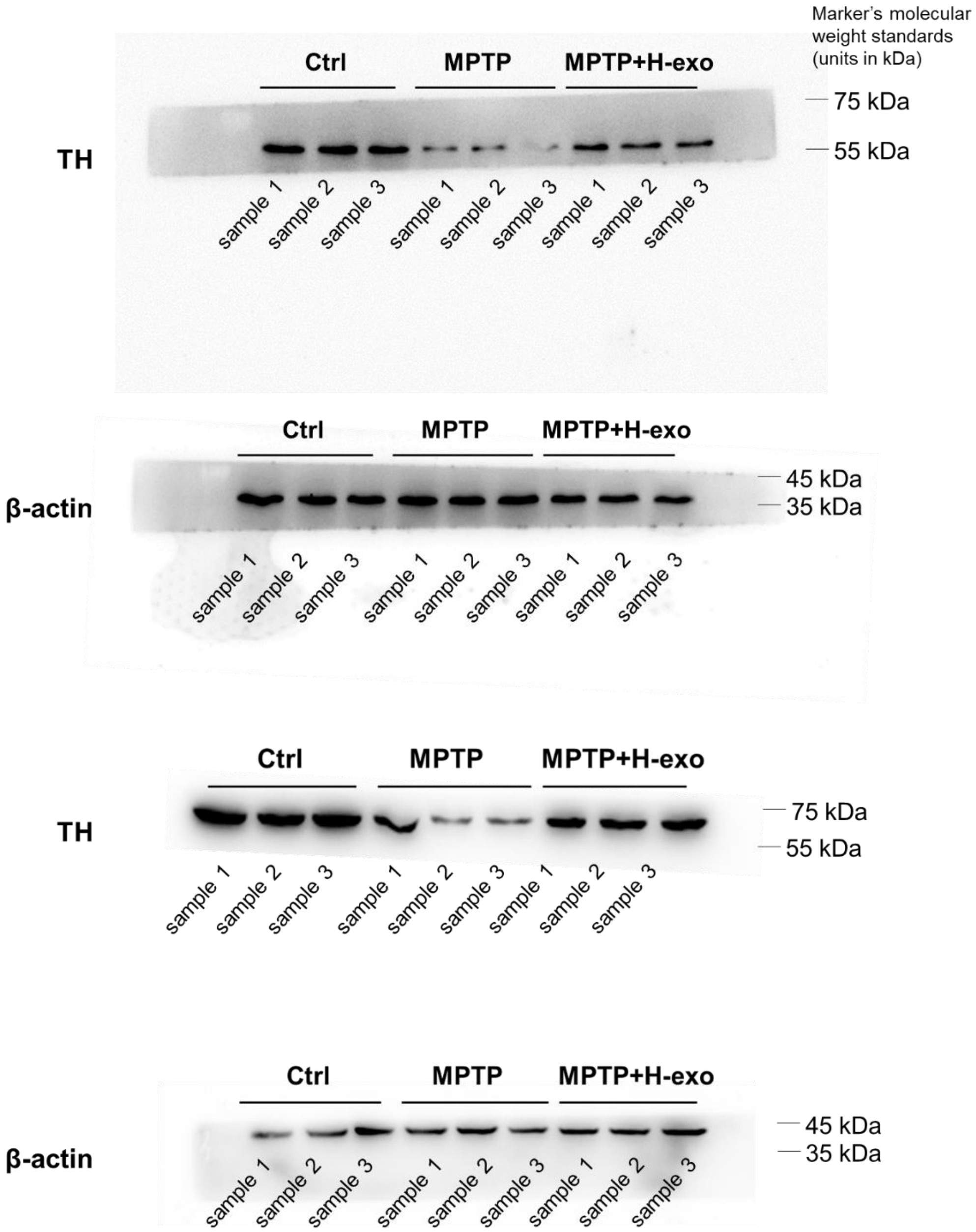

Full Unedited Gel/Blot for Figure 3G

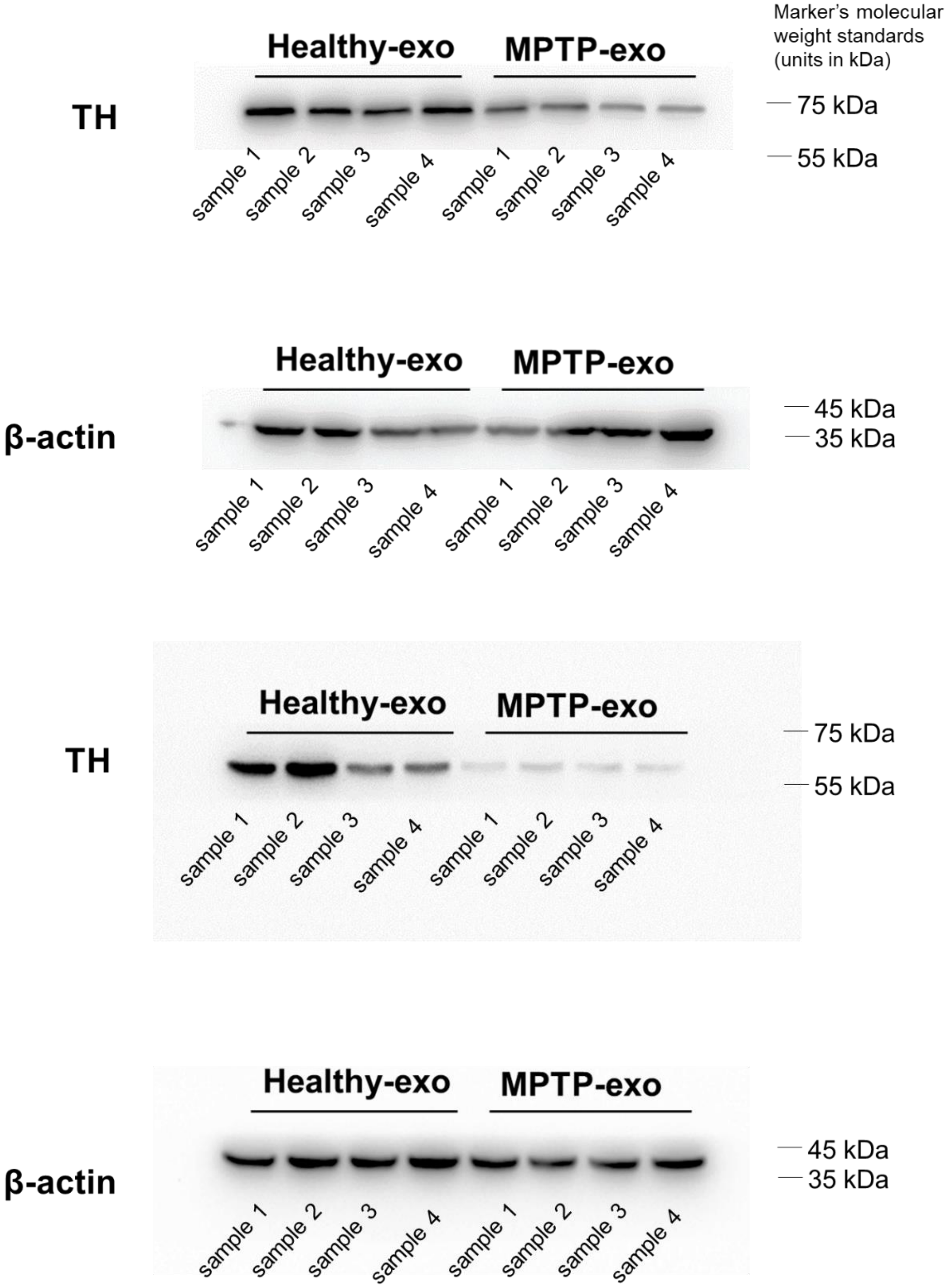

Full Unedited Gel/Blot for Figure 4J

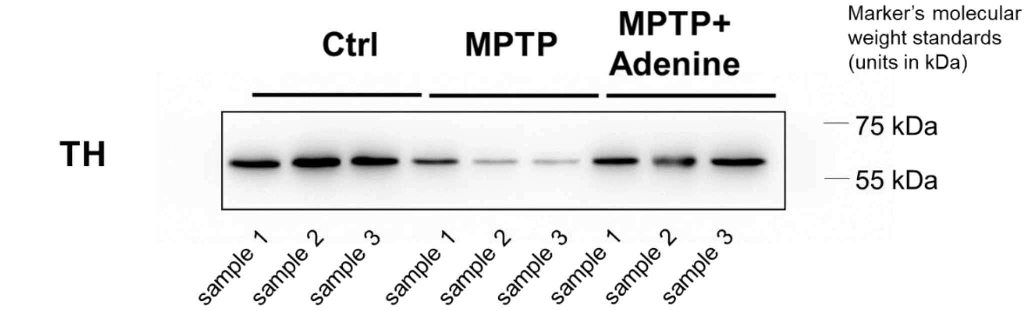

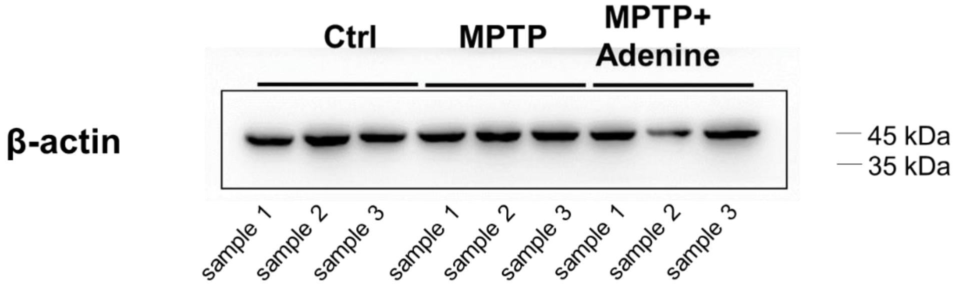

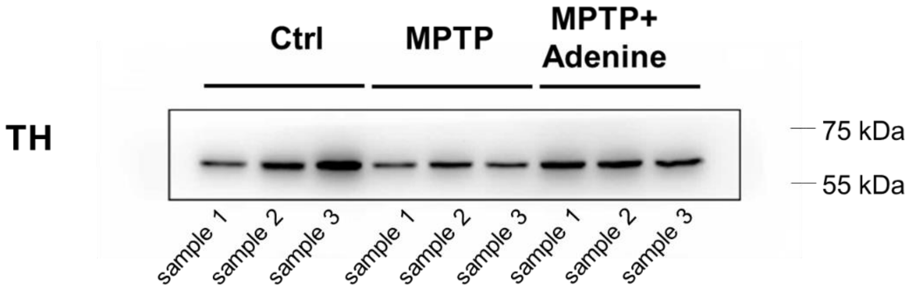

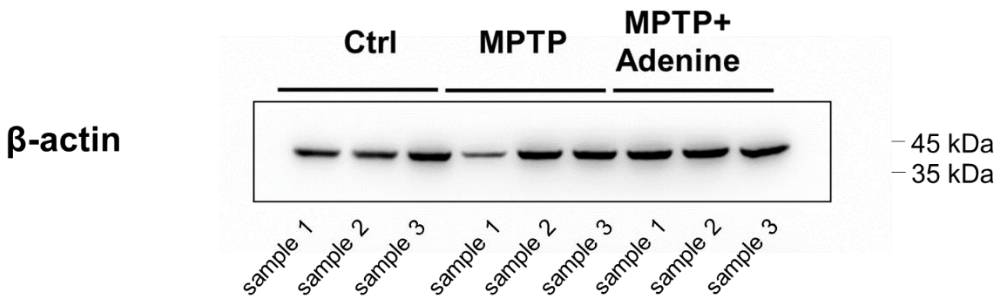

Full Unedited Gel/Blot for Figure 5G

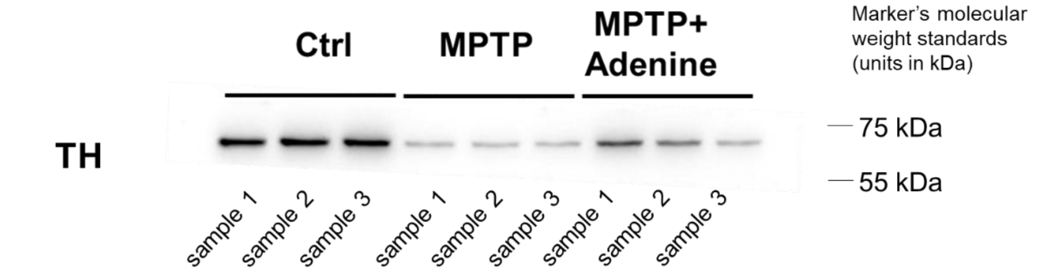

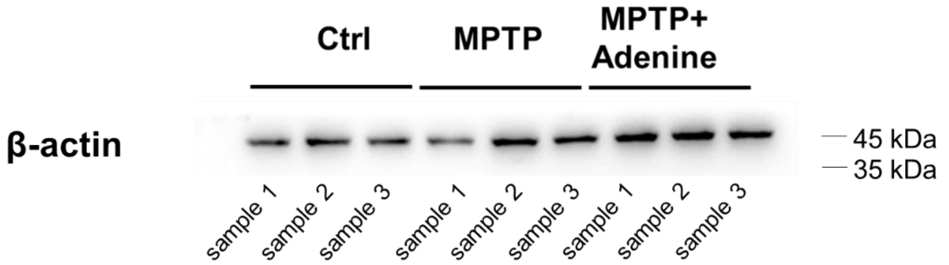

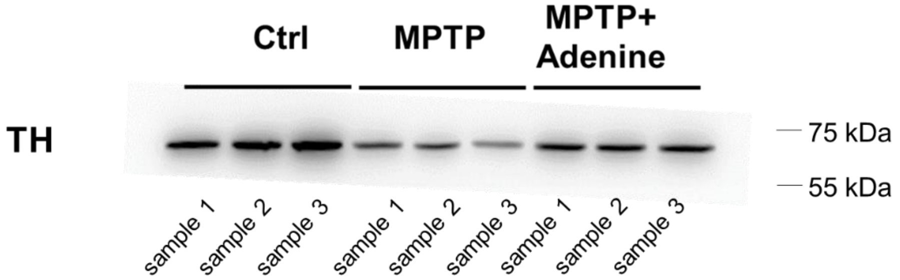

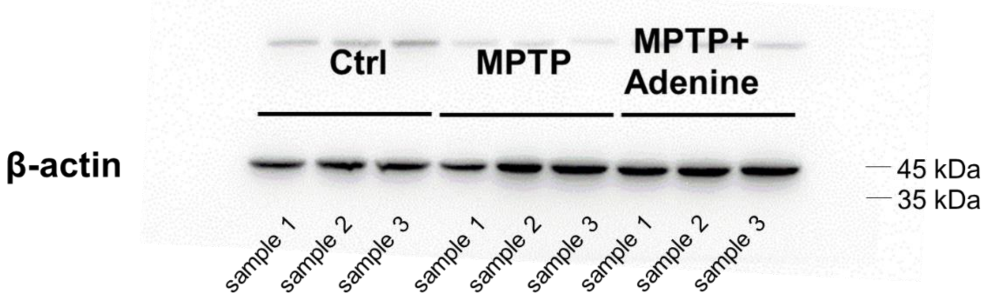

Full Unedited Gel/Blot for Figure 6A

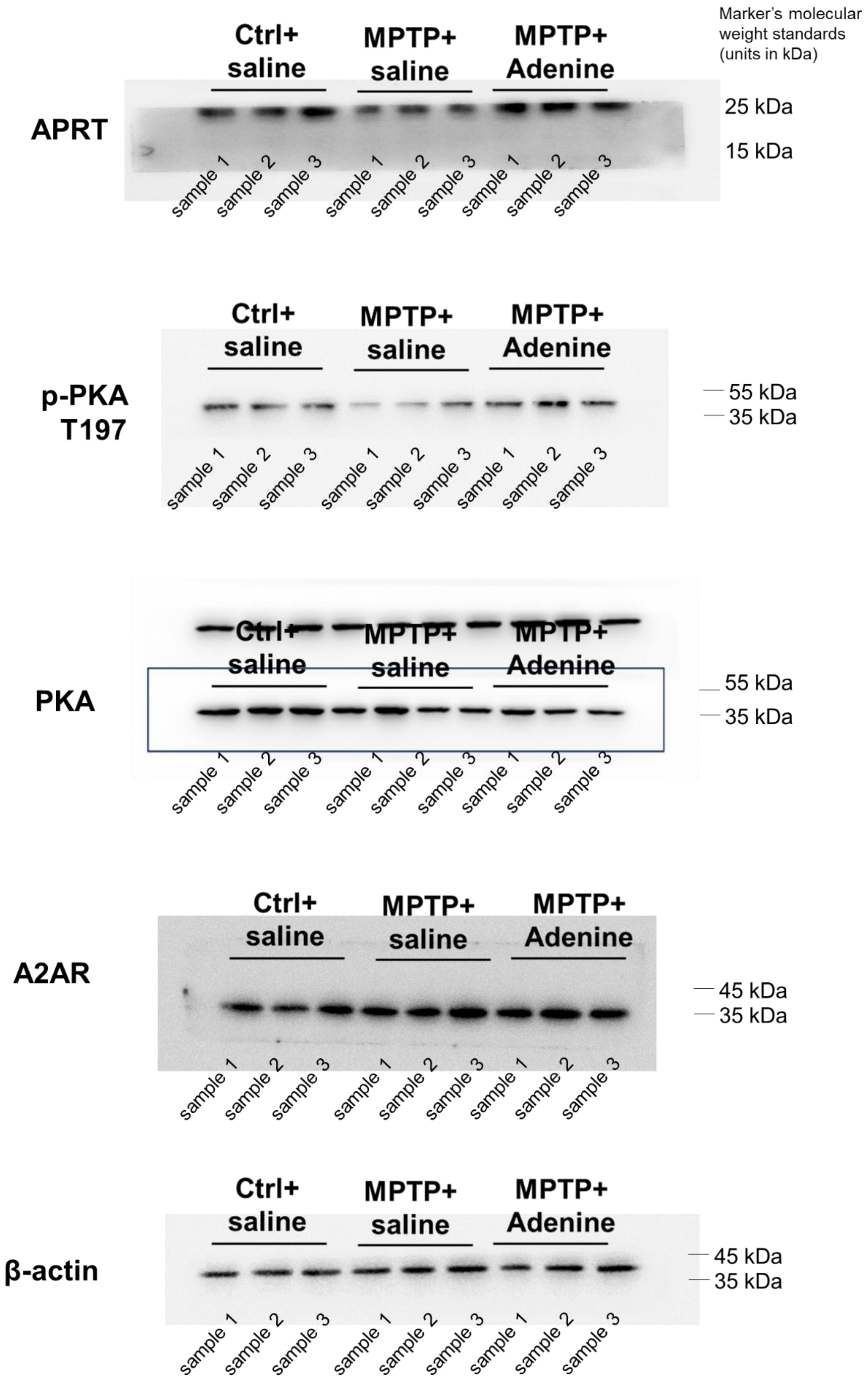

Full Unedited Gel/Blot for Figure 6F

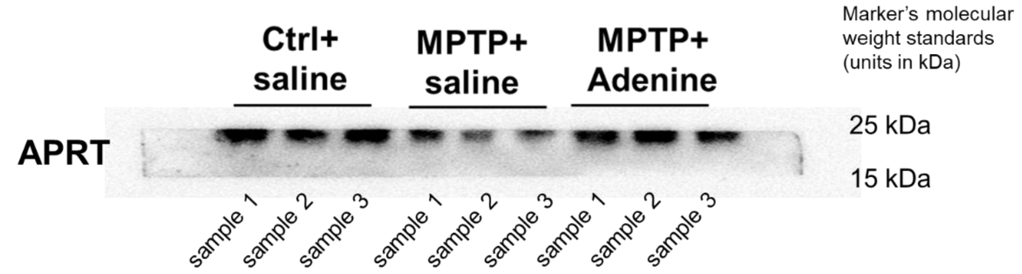

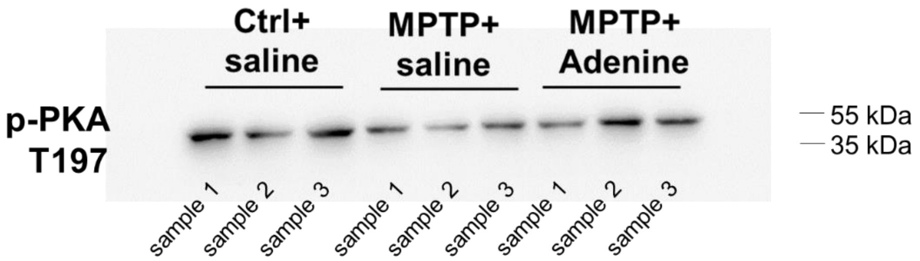

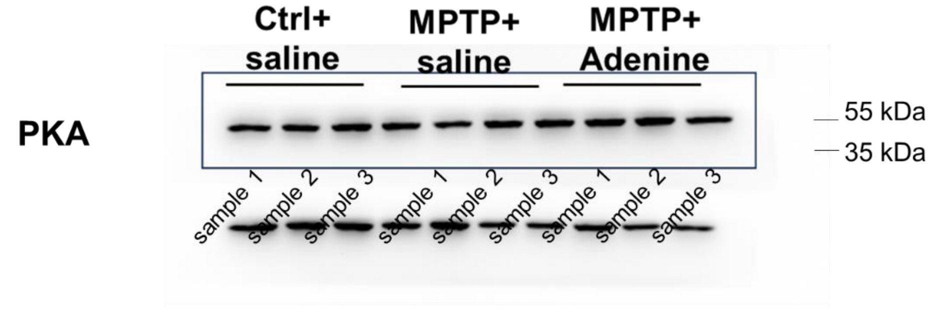

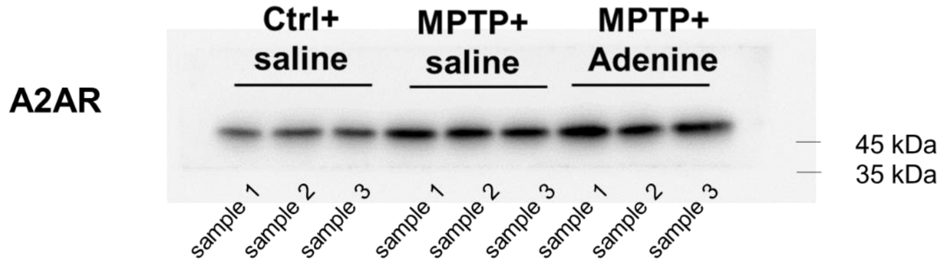

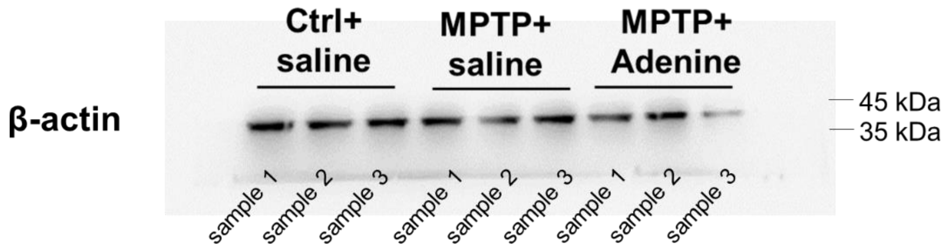

Supplement: Supplementary file 1 — Figure S1. Full unedited gel/blot for Figures. [file CNS-31-e70331-s001.zip › cns70331-sup-0002-Revised Supplemental Files -revision 2.pdf]
